# Functionalized Biomimetic Nanoparticles Targeting the IL‐10/IL‐10Rα/Glycolytic Axis in Synovial Macrophages Alleviate Cartilage Degeneration in Osteoarthritis

**DOI:** 10.1002/advs.202504768

**Published:** 2025-05-02

**Authors:** Wenwei Li, Yang Liu, Ming Wei, Zhichao Yang, Zhaoyu Li, Zezhong Guo, Liang Yan, Yang Lu, Hao Tang, Bofeng Li, Wei Huang

**Affiliations:** ^1^ Department of Orthopedics Centre for Leading Medicine and Advanced Technologies of IHM The First Affiliated Hospital of USTC Division of Life Sciences and Medicine University of Science and Technology of China Hefei Anhui 230001 China; ^2^ School of Medicine Anhui University of Science and Technology Huainan Anhui 232000 China; ^3^ Department of Orthopedics The First Affiliated Hospital of Anhui Medical University Hefei Anhui 230001 China; ^4^ Department of Medical Oncology The First Affiliated Hospital of USTC Division of Life Sciences and Medicine University of Science and Technology of China Hefei Anhui 230001 China; ^5^ Institute of Health and Medicine Hefei Comprehensive National Science Center Hefei Anhui 230001 China

**Keywords:** ferroptosis, glycolysis, HIF‐1α, IL‐10Rα, MOF, osteoarthritis

## Abstract

Osteoarthritis (OA) is a low‐grade inflammatory disease that is highly associated with severe hyperplasia of the synovial membrane and the degeneration of cartilage. Interleukin‐10 (IL‐10), has been extensively studied, while its receptor, IL‐10Rα, has not been widely mentioned in the context of OA. A significant difference is found in the expression of IL‐10Rα in synovial macrophages from normal and OA patients, along with a marked increase in the glycolytic activity of synovial macrophages. In IL‐10Rα^Lysm^ OA mice, the specific deficiency of IL‐10Rα exacerbated the progression of OA. Mechanistically, hypoxia‐inducible factor‐1α (HIF‐1α) is identified as a key transcription factor, and its inhibition significantly weakened the glycolytic process. Additionally, differences in ferroptosis of chondrocytes are observed. After co‐culturing the two types of cells in vitro, a significant connection is found between the glycolytic state of synovial macrophages and the ferroptosis of chondrocytes. To achieve targeted therapy, MI@UN, a biomimetic nanoparticle encapsulating NO‐prednisolone in UIO‐66‐NH_2_, surface‐modified with IL‐10, and coated with macrophage membranes (MM), is developed. It significantly slows osteoarthritis progression in mice. This offers new insights into OA pathogenesis, highlighting IL‐10Rα as a therapeutic target and supporting MI@UN's translational use for OA treatment.

## Graphical abstract

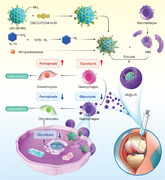

## 1 Introduction

Osteoarthritis (OA) is a chronic inflammatory condition characterized by synovitis and degenerative changes in articular cartilage, with clinical manifestations including pain and functional loss.^[^ ^1^ ^]^ In the early stages of OA, synovial tissue inflammation often precedes changes in bone and cartilage structure and is associated with disease progression.^[^ ^2^, ^3^, ^4^ ^]^ The synovial environment in OA patients is often characterized by partial oxygen deprivation, a condition exacerbated by inflammatory responses and limited joint motion, leading to inadequate vascular supply and subsequent hypoxic conditions.^[^ ^5^ ^]^ Under these hypoxic conditions, hypoxia‐inducible factor‐1α (HIF‐1α) plays a central role in cellular adaptation. HIF‐1α not only induces glycolysis by promoting the expression of glycolytic enzymes but also drives the synthesis of proinflammatory cytokines.^[^ ^6^, ^7^ ^]^ Within the confined joint cavity, synovial macrophages primarily rely on glycolysis for energy production.^[^ ^8^, ^9^ ^]^ Dysregulated energy metabolism is one of the key risk factors in OA.^[^ ^10^ ^]^ These mechanisms suggest that synovial hypoxia may exacerbate OA progression and symptoms by enhancing glycolysis and inflammation. Therefore, targeting excessive glycolysis, which is associated with alleviating synovial hypoxia, may provide a therapeutic strategy to mitigate OA‐related symptoms.

IL‐10 is a significant cytokine that helps to reduce inflammation and regulates immune responses.^[^ ^11^ ^]^ Previous studies showed that IL‐10 could inhibit lipopolysaccharide (LPS)‐induced glycolysis.^[^ ^12^ ^]^ IL‐10 exerts its effects by binding to the IL‐10 receptor, which consists of IL‐10Rα and IL‐10Rβ.^[^ ^13^, ^14^ ^]^ The primary function of IL‐10Rα is to bind IL‐10 and initiate its activity, with receptor assembly beginning with the binding of IL‐10 to IL‐10Rα, followed by the engagement of IL‐10Rβ, allowing the formation of a signaling complex.^[^ ^15^ ^]^ This highlights the importance of IL‐10Rα in this cascade reaction. However, the relationship between IL‐10Rα and HIF‐1α remains unclear.

Ferroptosis is a form of iron‐dependent, non‐apoptotic cell death characterized by the accumulation of lipid peroxidation products and reactive oxygen species (ROS).^[^ ^16^ ^]^ Accumulating evidence indicates that OA is closely associated with ferroptosis in chondrocytes, and this process is accelerated under inflammatory conditions, thereby exacerbating OA progression.^[^ ^17^, ^18^ ^]^ Acyl‐CoA synthetase long‐chain family member 4 (ACSL4) is a key biomarker and promoter of ferroptosis. It catalyzes the incorporation of polyunsaturated fatty acids into membrane phospholipids, thereby accelerating lipid peroxidation and the production of malondialdehyde (MDA).^[^ ^19^, ^20^ ^]^ Glutathione peroxidase 4 (GPX4) is a critical regulator of ferroptosis; its reduced activity leads to impaired detoxification of lipid peroxides.^[^ ^21^ ^]^ Ferroptosis in chondrocytes involves abnormalities in iron metabolism, lipid peroxidation, and inactivation of GPX4.^[^ ^22^, ^23^ ^]^ Collectively, these findings highlight the strong correlation between OA and chondrocyte ferroptosis.

The joint cavity is a closed environment,^[^ ^24^ ^]^ and the presence of synovial fluid provides the basis for indirect contact between cells. In OA, the interaction between synovial macrophages and chondrocytes remains unclear. Although similar reports exist,^[^ ^25^ ^]^ the underlying mechanisms are still poorly understood. According to the previous summary, there must be some connection between glycolysis of synovial macrophages and ferroptosis of chondrocytes. In the synovial fluid of OA patients, 55% of cytokines are produced by synovial cells, of which 39% are exclusive to these cells and not expressed by chondrocytes.^[^ ^26^ ^]^ This further indicates that there is a certain degree of crosstalk between synovial macrophages and chondrocytes. Cell–cell crosstalk also plays an important role in OA. Exploring the connection between them will help us to better understand the pathogenesis of OA.

Lately, nanoparticles encapsulated with cell membranes have become a promising therapeutic strategy.^[^ ^27^, ^28^ ^]^ These cell membrane‐coated nanoparticles inherit traits from their parent cells, including targeted delivery for efficacy, and demonstrate superior therapeutic outcomes.^[^ ^29^, ^30^, ^31^ ^]^ In order to achieve better therapeutic effects, we intend to adopt more effective targeted delivery methods. We then developed the MI@UN nanotherapeutic system, encapsulating NO‐prednisolone into UIO‐66‐NH_2_, modifying UIO‐66‐NH_2_ with IL‐10 through click chemistry, and finally enveloping this molecule with macrophage membranes (MM). NO‐prednisolone was used to stimulate the release of IL‐10, and the UIO‐66‐NH_2_ was used to prolong the NO‐prednisolone administration time and maintain the drug concentration. We then analyzed the characteristics of these nanoparticles, conducted biodistribution and pharmacodynamic experiments in mice, and assessed the inhibition of glycolysis in the synovium and ferroptosis in articular cartilage, validating the therapeutic effect on OA. According to our research results, this treatment can block excessive glycolysis in the synovium and the subsequent inflammatory cascade, successfully alleviating damage to chondrocytes, with significant anti‐inflammatory and reparative effects.

## 2 Results

### 2.1 Excessive Glycolysis in Synovial Tissue and Ferroptosis in Chondrocytes in OA Patients and OA Model Mice

**Figure 1:**
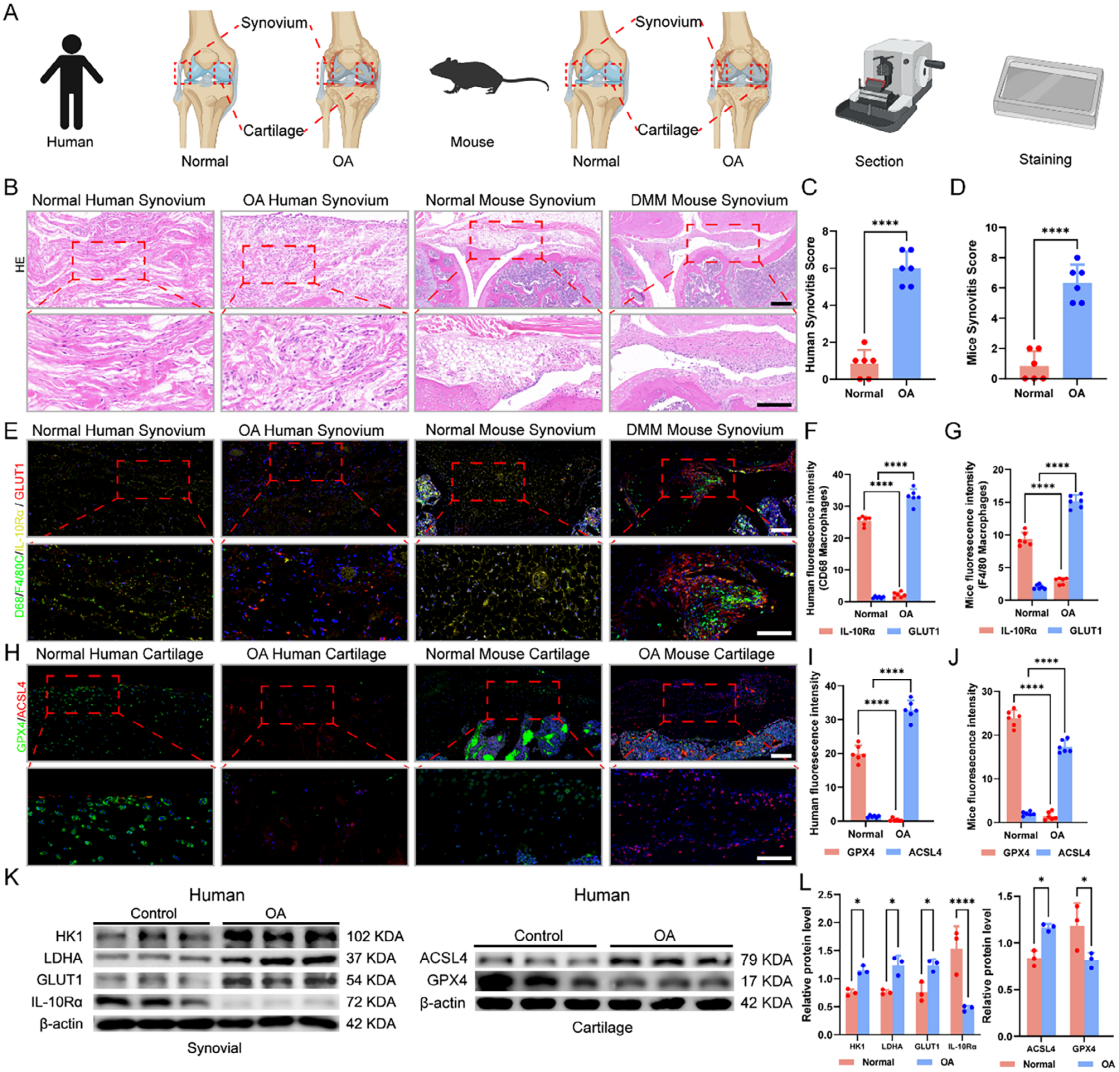
Differences in synovial and cartilage tissues between patients with OA and mice with OA. A) Human and murine synovial and cartilage tissues were extracted for subsequent experiments. B) HE staining of synovial tissues from healthy controls or OA patients and mice with OA. Scale bar: 100 µm. C) Quantification of the synovitis score of normal and osteoarthritic human synovial tissues. D) Quantification of the synovitis scores of normal and DMM model mouse synovial tissues. E) Immunofluorescence (IF) staining of synovial tissues from healthy controls or OA patients and mice with normal or OA. Scale bar: 100 µm. F) Quantification of IF staining of normal or osteoarthritic human synovial tissues with CD68 labeling of macrophages. G) Quantification of IF staining of normal and DMM model mouse synovial tissues with F4/80 labeling of macrophages. H) IF staining of cartilage tissues from healthy controls or OA patients and mice with normal or OA. Scale bar: 100 µm. I) Quantification of IF staining of normal or osteoarthritic human cartilage tissues. J) Quantification of IF staining of normal and DMM model mouse cartilage tissues. K) WB for glycolytic markers in synovial tissues and ferroptosis markers in cartilage tissues from healthy controls or OA patients. L) Quantification of WB data for normal or osteoarthritic human synovial and cartilage tissues. ^*^ *p* < 0.05, ^**^ *p* < 0.01, ^***^ *p* < 0.001, ^****^ *p* < 0.001, ns indicates not significant.

To investigate the role of synovial macrophage glycolysis and chondrocyte ferroptosis in the development of OA, we initially examined synovial conditions in humans and mice (**Figure** **1**A). We noted significant synovial hyperplasia and extensive cellular infiltration in human osteoarthritic synovial tissues. Similarly, the synovium of mice in the destabilization of the medial meniscus (DMM) model mirrored the human condition, with DMM intensifying the macrophage count within the synovial tissue and disrupting its original architecture. Notably, minimal synovial proliferation was observed in normal human and mouse synovial tissues, with clear synovial architecture visible (Figure 1B). Consistent with previous results,^[^ ^28^ ^]^ the synovitis score of human osteoarthritic synovial tissues was significantly greater than that of normal controls (Figure 1C), and a similar pattern was observed in mice (Figure 1D). Building on our previous assumptions, we assessed the levels of IL‐10Rα and the principal glycolytic initiator protein Glucose Transporter 1 (GLUT1) in both human and murine synovial tissues. Macrophages were labeled with CD68 or F4/80, as expected, IL‐10Rα was highly expressed in normal human and mouse synovial tissues, and GLUT1 was highly expressed in the human osteoarthritic synovium and DMM model mouse synovium (Figure 1E–G). The concurrent changes in IL‐10Rα and GLUT1 suggest a potential link between IL‐10Rα and glycolysis. We subsequently examined the expression of the ferroptosis markers GPX4 and ACSL4 in human and mouse cartilage. Normal human and control mouse cartilage presented high expression of GPX4 and low expression of ACSL4, whereas osteoarthritic human and DMM model mouse cartilage tissues presented the opposite results, identifying ferroptosis in the articular cartilage of individuals or mice with OA (Figure 1H–J). To further validate our experimental results, we extracted proteins from human synovial and cartilage tissues from the control and OA groups. In the synovium, we verified the presence of IL‐10Rα and several key glycolytic proteins, such as Hexokinase 1 (HK1), Lactate Dehydrogenase A (LDHA), and GLUT1. The Western blot (WB) results revealed that, compared with those in the control samples, the osteoarthritic synovium exhibited excessive glycolysis and significant suppression of the expression of IL‐10Rα. Concurrently with synovial glycolysis, osteoarthritic cartilage expresses signals of ferroptosis (Figure 1K,L). In summary, these findings indicate that macrophages aggregate in osteoarthritic synovial tissues and produce excessive glycolysis, with IL‐10Rα likely involved in this process, and that excessive glycolysis may induce ferroptosis in chondrocytes.

### 2.2 IL‐10Rα Deficiency in Macrophages Increases Glycolysis in the Osteoarthritic Synovium and Intensifies Chondrocyte Ferroptosis, Promoting OA Progression

**Figure 2:**
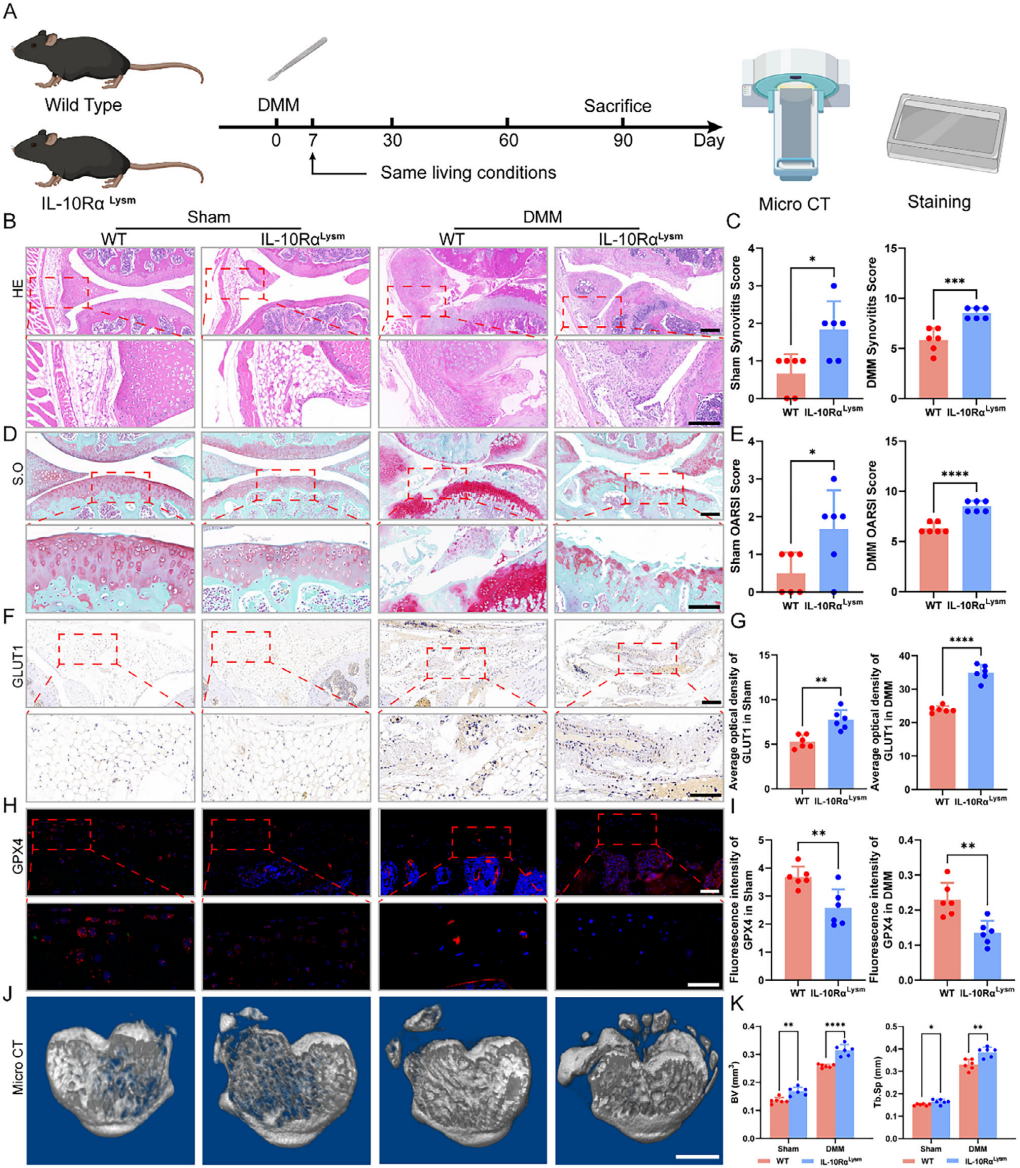
Validation of the impact of IL‐10Rα on OA via generation of IL‐10Rα^Lysm^ mice. A) DMM was performed on IL‐10Rα^Lysm^ mice and their littermate WT, and the survival of the mice was observed 7 days post‐surgery before random grouping. The mice were provided with the same living conditions, and knee joint samples were harvested for subsequent experiments after 90 days. B) HE staining of synovial tissue from the sham and DMM groups of mice. Scale bar: 100 µm. C) Quantification of the synovitis score of the synovial tissue from the sham and DMM groups of mice. D) S.O staining of cartilage tissue from the sham and DMM groups of mice. Scale bar: 100 µm. E) The OARSI score was quantified for mice in the sham and DMM groups. F,G) IHC staining and quantification of synovial tissue from the sham and DMM groups of mice. Scale bar: 100 µm. H,I) IF staining and quantification of cartilage tissue from the sham and DMM groups of mice. Scale bar: 100 µm. J,K) Top Micro‐CT view of the subchondral bone and quantitative analysis of the BV and Tb.Sp in the sham and DMM groups of mice (*n* = 6). Scale bar: 1 mm. ^*^ *p* < 0.05, ^**^ *p* < 0.01, ^***^ *p* < 0.001, ^****^ *p* < 0.001, ns indicates not significant.

To explore the function of IL‐10Rα in macrophages and chondrocytes, we generated bone marrow‐specific IL‐10Rα knockout (IL‐10Rα^Lysm^) mice (Figure S1A, Supporting Information) and conducted DMM on 3‐month‐old IL‐10Rα^Lysm^ mice to elicit osteoarthritis and their littermate wild type (WT) controls (**Figure** **2**A). IL‐10Rα^Lysm^ mice presented increased pathogenesis at 12 weeks post‐surgery. Hematoxylin‐eosin (HE) staining revealed significant synovial hyperplasia and structural disruption, with markedly higher synovitis scores than those of the WT mice (Figure 2B,C). Cartilage damage was more severe, and safranin (S.O). staining revealed that the IL‐10Rα^Lysm^ mice had some degree of cartilage damage even without any treatment. Compared with the WT mice, the IL‐10Rα^Lysm^ mice presented an inherently uneven cartilage surface with no notable synovial hyperplasia; however, post‐DMM modeling, the degree of joint cartilage damage in the IL‐10Rα^Lysm^ mice was greater than that in their littermates, with IL‐10Rα deficiency exacerbating cartilage wear (Figure 2D). Additionally, the OARSI score was significantly greater in the DMM postsurgery IL‐10Rα^Lysm^ group (Figure 2E). We also assessed glycolysis in synovial macrophages via immunohistochemical (IHC) staining, which revealed that the expression of GLUT1, a key glycolytic molecule, was greater in the IL‐10Rα^Lysm^ mice than in the WT mice under both normal and DMM conditions, indicating that IL‐10Rα deletion induced intense glycolysis in synovial macrophages (Figure 2F,G). Similarly, we assessed the key ferroptosis regulator GPX4 in chondrocytes, and a difference was observed even before DMM and was significantly magnified after DMM (Figure 2H,I). We also performed Micro‐CT scanning on the joints of the mice to determine changes in subchondral bone, revealing an increase in osteophyte formation after DMM, especially in the IL‐10Rα^Lysm^ mice. By sectioning the subchondral bone, we observed that the DMM mice had hardened subchondral bone, which was particularly pronounced in the IL‐10Rα^Lysm^ mice. Quantitative analysis of the BV, BV/TV, trabecular number (Tb.N), and trabecular separation (Tb.Sp) was conducted via software. The parameters BV, BV/TV, and Tb.N showed significant decreases in the DMM group, with an even more pronounced decline in the IL‐10Rα^Lysm^ group, whereas the results for Tb.Sp were the opposite (Figure 2J,K; Figure S1B,C, Supporting Information). In summary, the deletion of IL‐10Rα induced increased glycolysis in synovial macrophages, exacerbated ferroptosis in chondrocytes, and further advanced the development of OA.

### 2.3 Potential Mechanisms of Action of IL‐10Rα

**Figure 3:**
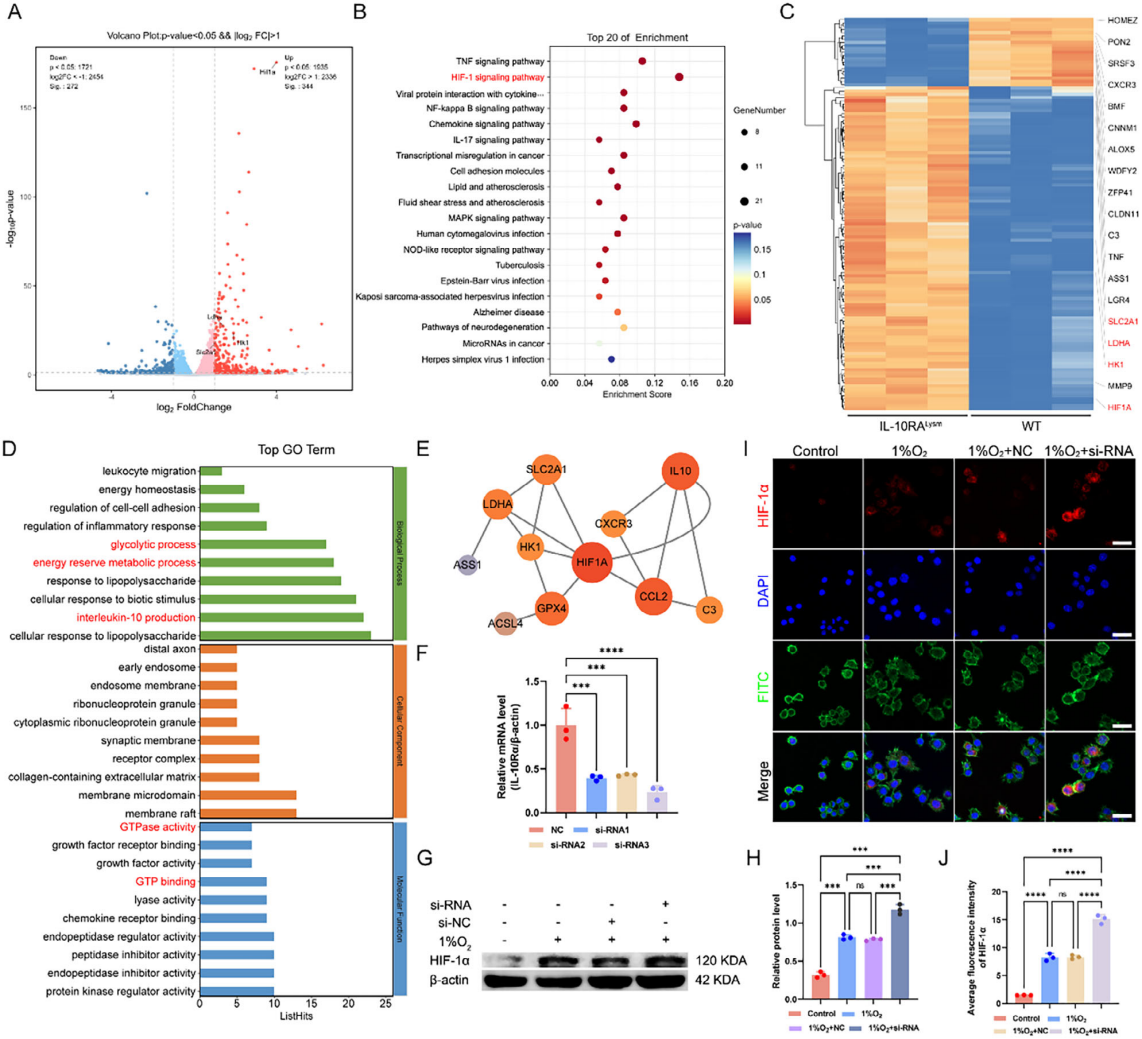
RNA sequencing to identify potential downstream pathways and molecules of IL‐10Rα. A) Volcano plot displaying differentially expressed genes. B) The top 20 enriched pathways from the KEGG analysis. C) Heatmap representing the differential expression of genes related to cellular metabolism. D) The top 30 genes from the GO analysis that were differentially expressed. E) PPI network diagram showing proteins that interact with HIF‐1α. F) qPCR screening to identify the most effective siRNA. G,H) WB experiments to validate the changes in the expression of HIF‐1α following IL‐10Rα knockdown, with quantification graph. I,J) IF experiments to validate the changes in HIF‐1α expression following IL‐10Rα knockdown, with quantification graph. Scale bar: 20 µm. ^*^ *p* < 0.05, ^**^ *p* < 0.01, ^***^ *p* < 0.001, ^****^ *p* < 0.001, ns indicates not significant.

To validate the potential mechanisms of action of IL‐10Rα, we sorted primary synovial macrophages from IL‐10Rα^Lysm^ and littermate WT mice post‐DMM and performed RNA‐seq on the obtained primary macrophages to identify downstream pathways and differentially expressed genes. The principal component analysis (PCA) results indicated good sample reproducibility (Figure S2A, Supporting Information). Total of 306 differentially expressed genes were identified, with 111 downregulated genes and 195 upregulated genes (Figure S2B, Supporting Information). Volcano plot analysis revealed that HIF‐1α was the most significant gene involved in glucose metabolism, and other glycolysis‐related genes, such as LDHA, HK1, and GLUT1, were also differentially expressed (**Figure** **3**A). Kyoto encyclopedia of genes and genomes (KEGG) revealed that the HIF‐1α signaling pathway was the most enriched pathway related to energy metabolism (Figure 3B). Heatmaps revealed that all the aforementioned genes (LDHA, HK1, Solute carrier family 2 member 1 (SLC2A1), and HIF‐1α) were differentially expressed between the IL‐10Rα^Lysm^ and WT groups (Figure 3C). Gene ontology (GO) analysis revealed that glucose metabolism, energy metabolic processes, and IL‐10 production were among the top 10 biological processes, and GTP‐related energy metabolism was also associated with molecular functions (Figure 3D). Protein‒protein interaction (PPI) analysis revealed that HIF‐1α could interact with IL‐10, as well as with the glycolysis‐related proteins HK1, LDHA, and SLC2A1 and the ferroptosis‐related proteins GPX4 and ACSL4 (Figure 3E). We selected the most effective siRNA, siRNA‐3, for further experiments via qPCR screening (Figure 3F). We subsequently verified the connection between IL‐10Rα and HIF‐1α. We knocked down IL‐10Rα in macrophages and found that under hypoxic conditions, the expression of HIF‐1α significantly increased, and this increase was more pronounced with the knockdown of IL‐10Rα (Figure 3G,H), indicating a potential link between IL‐10Rα and HIF‐1α. IF staining of macrophages revealed that the expression of HIF‐1α was consistent with the WB results, with hypoxia promoting HIF‐1α expression and IL‐10Rα knockdown exacerbating this effect (Figure 3I,J). In summary, the sequencing results aligned with our expectations for OA, and we identified the potential regulatory molecule HIF‐1α that affects glycolysis, providing a foundation for our subsequent experiments.

### 2.4 Validation of the Macrophage Glycolytic Pathway

**Figure 4:**
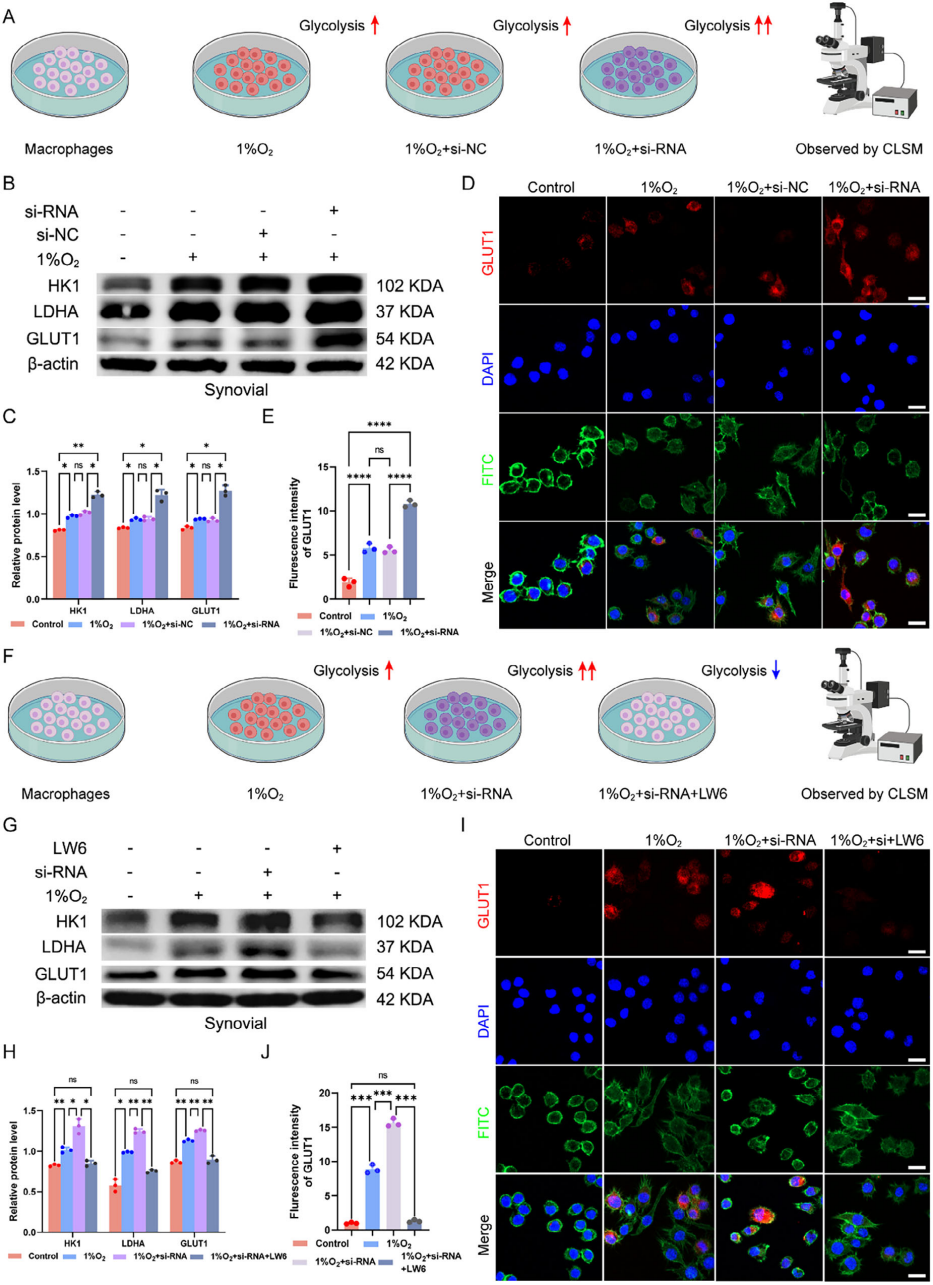
Validation of the potential downstream pathways of macrophage glycolysis. A) Schematic diagram of the process used to induce macrophage glycolysis and verify the changes in glycolytic proteins. B,C) Glycolysis was induced in macrophages, followed by the addition of IL‐10Rα siRNA, and glycolytic‐related proteins were detected via WB analysis, along with quantification graph. D,E) After macrophage glycolysis was induced, IF experiments were conducted to detect the changes in GLUT1 following the knockdown of IL‐10Rα, and the results were quantified. Scale bar: 20 µm. F) Schematic diagram of the process involving the induction of macrophage glycolysis and the addition of a HIF‐1α inhibitor to verify the changes in glycolytic proteins. G,H) Building on previous experiments, a HIF‐1α inhibitor was added, and WB was used to detect glycolytic‐related proteins, along with quantification graph. I,J) IF experiments were used to verify the changes in GLUT1 after the addition of a HIF‐1α inhibitor, along with quantification graph. Scale bar: 20 µm. ^*^ *p* < 0.05, ^**^ *p* < 0.01, ^***^ *p* < 0.001, ^****^ *p* < 0.001, ns indicates not significant.

On the basis of the sequencing results, we utilized a macrophage to induce glycolysis and examined the impact of IL‐10Rα knockdown on this process (**Figure** **4**A). After inducing glycolysis in macrophages, we assessed the expression of the glycolytic proteins HK1, LDHA, and GLUT1. We observed that hypoxia induced glycolysis, and the knockdown of IL‐10Rα led to an even more robust glycolytic response (Figure 4B,C). IF further confirmed the effect of IL‐10Rα deletion on macrophage glycolysis. Compared with normoxic conditions, hypoxia stimulated greater expression of GLUT1, and the deletion of IL‐10Rα resulted in a significant increase in the expression of GLUT1 (Figure 4D,E). To validate the regulation of glycolysis by HIF‐1α, we knocked down IL‐10Rα, treated the cells with a HIF‐1α inhibitor, and then measured the expression of glycolytic proteins (Figure 4F). We repeated the previous experiments with the addition of the HIF‐1α inhibitor and assessed the expression of the glycolytic proteins HK1, LDHA, and GLUT1. We found that excessive glycolysis was alleviated by the addition of the HIF‐1α inhibitor LW6, as shown by the reduced expression of HK1, LDHA, and GLUT1 (Figure 4G,H). We also examined the fluorescence expression of GLUT1, and the results were consistent with those of the WB analysis, which revealed that LW6 reversed the increase in glycolysis (Figure 4I,J). We added IL‐10 to hypoxic macrophages and used qPCR to measure the expression of IL‐1β, TNF‐α, IL‐6, and IL‐10Rα. We discovered that the addition of IL‐10 could counteract the hypoxia‐induced increase in inflammatory factors after 24 h and increase the expression of IL‐10Rα after 48 h (Figure S3A, Supporting Information). These findings collectively indicate that the deletion of IL‐10Rα intensifies the degree of glycolysis and that the addition of the HIF‐1α inhibitor LW6 reverses excessive glycolysis, IL‐10 could increase the expression of IL‐10Rα, confirming the relationship among IL‐10, IL‐10Rα and HIF‐1α.

### 2.5 Validation of Chondrocyte Ferroptosis in a Conditioned Medium (CM) Coculture System

**Figure 5:**
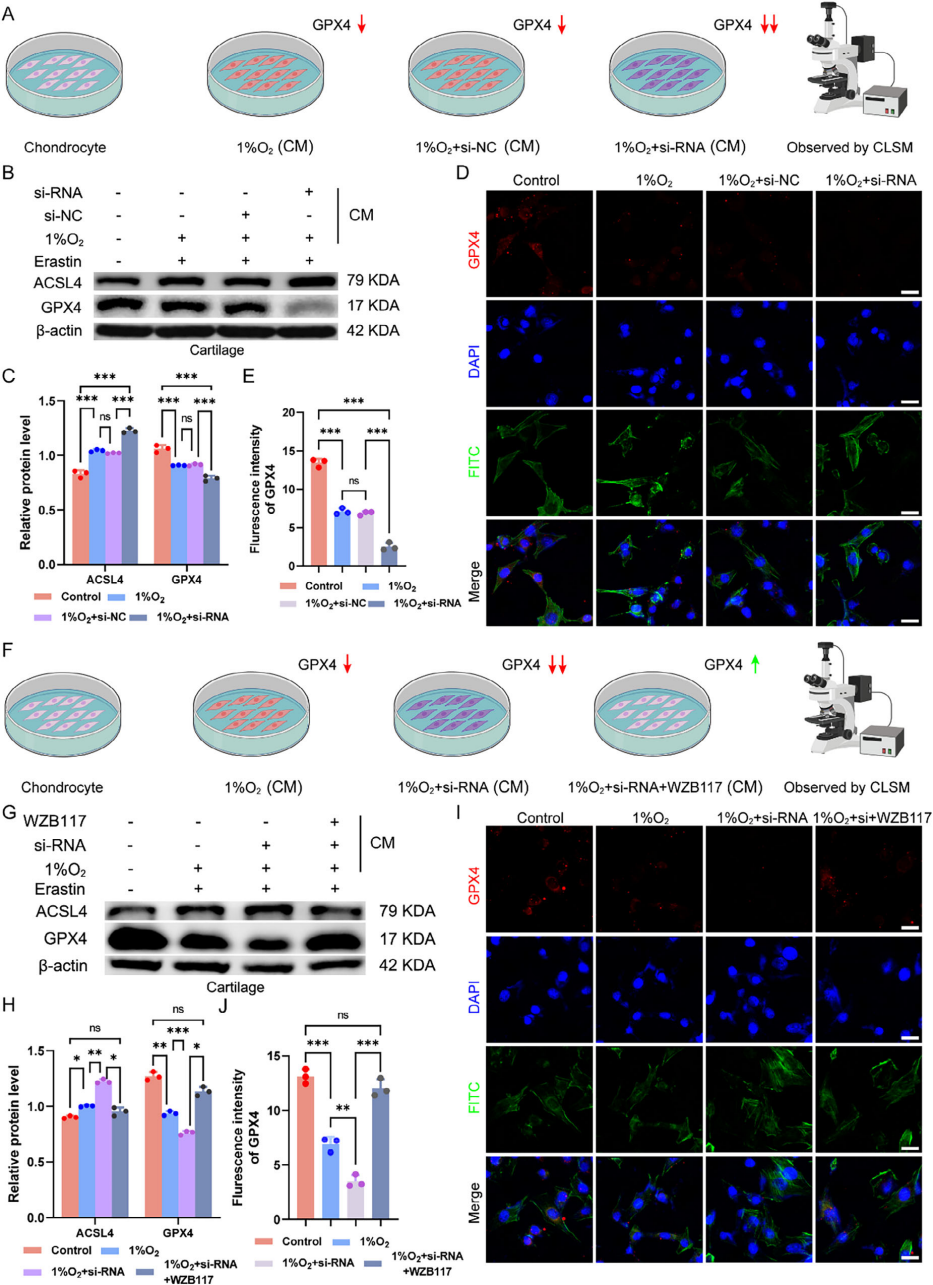
Coculture with CM was used to validate cell‒cell crosstalk. A) Schematic diagram of the process for coculturing chondrocytes with macrophage supernatant. B,C) Chondrocytes were treated with ferroptosis inducers and the corresponding CM, and the expression of ferroptosis‐related proteins in chondrocytes was determined, along with quantification graph. D,E) After ferroptosis was induced in chondrocytes, IL‐10Rα was knocked down, and changes in GLUT1 were determined via IF experiments, along with quantification graph. Scale bar: 20 µm. F) Schematic diagram of the process by which ferroptosis is induced in chondrocytes, and a glycolysis inhibitor was added to verify changes in ferroptosis‐related proteins. G,H) In previous experiments, a glycolysis inhibitor was added, and WB was used to assess ferroptosis‐related proteins, along with quantification graph. I,J) IF experiments were used to verify the changes in GLUT1 after the addition of a glycolysis inhibitor, along with quantification graph. Scale bar: 20 µm. ^*^ *p* < 0.05, ^**^ *p* < 0.01, ^***^ *p* < 0.001, ^****^ *p* < 0.001, ns indicates not significant.

To investigate the crosstalk between cells, we initially induced ferroptosis in chondrocytes via the inducer erastin and then cocultured these chondrocytes with the supernatant from macrophages. We then assessed ferroptosis in chondrocytes (**Figure** **5**A). WB analysis was conducted to evaluate the key proteins associated with ferroptosis, GPX4 and ACSL4. Our findings revealed that culture with CM led to notable changes in chondrocyte ferroptosis. The addition of erastin resulted in an increase in ACSL4 levels, and this increase was further exacerbated by CM from IL‐10Rα‐knockdown macrophages (Figure 5B,C). Similarly, IF experiments were performed to observe the effects of CM on chondrocyte ferroptosis. We found that the expression of GPX4 was nearly undetectable under the influence of erastin and CM (Figure 5D,E). Both the WB and IF results indicated the occurrence of crosstalk between macrophages and chondrocytes, and this interaction affected ferroptosis in chondrocytes. To further confirm the cellular crosstalk, we added a glycolysis inhibitor to the macrophage supernatant, and the CM was collected to culture the chondrocytes (Figure 5F). WB results indicated that the addition of the glycolysis inhibitor significantly suppressed ferroptosis, with expression of GPX4 markedly higher in the treated group than in the damaged group, while ACSL4 expression had the opposite effect (Figure 5G,H). IF analysis also yielded similar outcomes. After staining for GPX4, we observed that coculturing chondrocytes with CM containing WZB117 mitigated the effects of erastin, leading to an increase in expression of GPX4 (Figure 5I,J). To pinpoint the key molecule in macrophage glycolysis that induces ferroptosis in chondrocytes, we assayed macrophage supernatants and found significant lactate differences, positing it as the crucial molecule linking macrophage glycolysis to chondrocyte ferroptosis (Figure S4A, Supporting Information). Subsequent experiments where we cultured chondrocytes with these supernatants revealed that under hypoxia, IL‐10Rα knockdown markedly enhanced ferroptosis, an effect attenuated by lactate inhibition. This underscores the role of the macrophage glycolysis‐chondrocyte ferroptosis crosstalk (Figure S4B,C, Supporting Information). In vitro, we administered intra‐articular injections of a glycolysis inhibitor to WT and IL‐10Rα^Lysm^ mice after DMM. Joint sample collection and staining revealed a clearer synovial structure with significantly inhibited glycolysis (Figure S5A,B,E, Supporting Information). Cartilage damage was also alleviated, but was more severe in IL‐10Rα^Lysm^ mice (Figure S5C,D, Supporting Information). Additionally, expression of the ferroptosis marker ACSL4 was inhibited (Figure S5F, Supporting Information). In conclusion, by utilizing CM to mimic cellular interactions, we discovered the interplay between macrophages and chondrocytes and demonstrated that the glycolytic process in synovial macrophages can influence the progression of ferroptosis in chondrocytes.

### 2.6 Construction and Characterization of MI@UN

**Figure 6:**
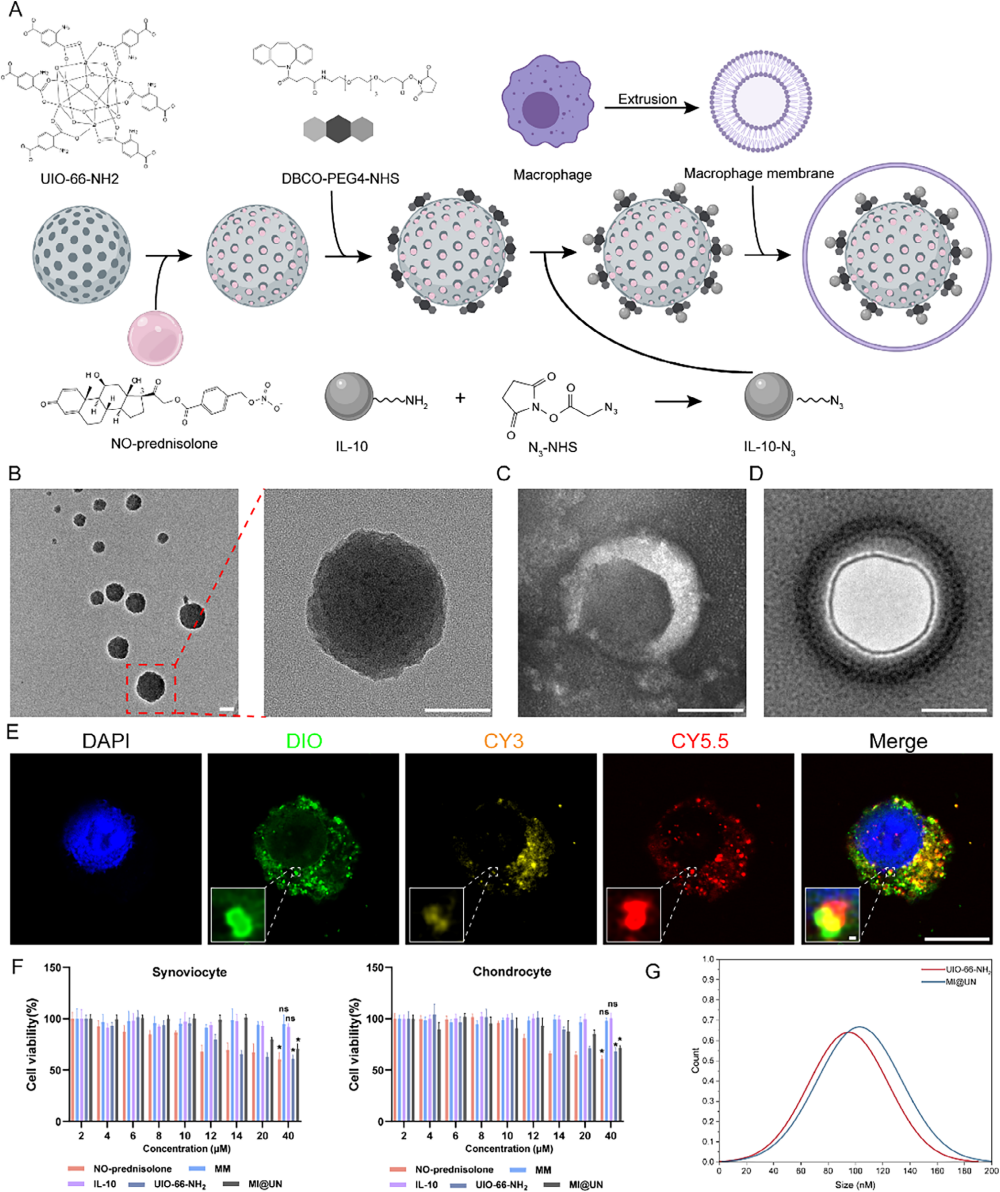
Construction and characterization of MI@UN. A) Schematic diagram of the components and fabrication process of MI@UN. B) TEM image showing the loose and porous structure of UIO‐66‐NH_2_. Scale bar: 50 nm. C) TEM image of the MM. Scale bar: 50 nm. D) TEM image of MI@UN. Scale bar: 50 nm. E) Confocal microscopy image of macrophages phagocytosing MI@UN, with nuclei stained with DAPI, MM stained with DIO, IL‐10 stained with CY3, and NO‐prednisolone stained with CY5.5. Scale bar: 20 µm. F) Cell viability of macrophages and chondrocytes after incubation with various concentrations of NO‐prednisolone, MM, IL‐10, UIO‐66‐NH_2_, and MI@UN. ^*^ *p* < 0.05, ns indicates not significant. G) Particle size distributions of UIO‐66‐NH_2_ (red) and MI@UN (blue).

Functionalized biomimetic nanoparticles for drug delivery were constructed. We encapsulated NO‐prednisolone within the pores of UIO‐66‐NH_2_. To modify UIO‐66‐NH_2_ with IL‐10, we employed a click chemistry reaction in which DBCO‐PEG4‐NHS was covalently bound to the amino groups on the surface of UIO‐66‐NH_2_, introducing DBCO moieties. The DBCO‐modified UIO‐66‐NH_2_ subsequently reacted with IL‐10‐N_3_ to form a stable triazole linkage. Finally, the nanoparticles were coated with MM to obtain our final product (**Figure** **6**A). The diameter of UIO‐66‐NH_2_ was ≈100 nm, and we could also see its porous structure, which provided the basis for sustained drug release (Figure 6B). TEM was also used to observe the cell membrane vesicles obtained by extruding the MM, under the TEM, MM vesicles appear as spherical structures with a hollow interior and a distinct lipid bilayer membrane (Figure 6C). The modified MOFs were combined with the MM and extruded to form the final product, which was then observed via TEM to assess its morphology and We can observe that modified MOFs is completely encapsulated within MM (Figure 6D). To verify the encapsulation of NO‐prednisolone and the conjugation of IL‐10, we used three different fluorescent dyes for labeling: CY3 for IL‐10, CY5.5 for NO‐prednisolone, and DIO‐Green for the MM. As shown in Figure 6E, the three dyes almost completely overlapped. In our in vitro study, the appearance of the MI@UN characteristic peak in the FTIR test results demonstrates the successful conjugation of IL‐10‐N_3_ and loading of NO‐prednisolone (Figure S6A, Supporting Information). HPLC analysis also confirms the successful loading of both NO‐prednisolone and IL‐10‐N_3_. To verify the loading efficiency of NO‐prednisolone, we measured its concentration in the solution before and after loading onto UIO‐66‐NH_2_. The difference in concentration before and after loading confirms the successful loading of NO‐prednisolone (Figure S6B, Supporting Information). The same method was used to test the loading of IL‐10‐N_3_, which also proved to be successful (Figure S6C, Supporting Information). Additionally, release experiments showed that IL‐10 was rapidly released within 12 h, while NO‐prednisolone was released over a period of 7 days (Figure S6D, Supporting Information). This finding aligns with our expectation that IL‐10 would be rapidly released to alleviate the inflammatory environment, whereas NO‐prednisolone would be slowly released to promote the production of IL‐10. ZETA potential measurements revealed that the surface of UIO‐66‐NH_2_ was initially negatively charged before loading. After the loading and encapsulation processes, the surface charge of MI@UN changed to positive (Figure S6H, Supporting Information). Figure 6E also displays the phagocytosis of MI@UN by macrophages. The macrophages exhibited excellent uptake of MI@UN, with the cytoplasm filled with green MM vesicles. Similarly, we observed the uptake of MI@UN by chondrocytes (Figure S6F, Supporting Information), noting that the number of vesicles inside chondrocytes was much lower and that the uptake of MI@UN was significantly lower than that by macrophages. To further verify the targeting ability of MI@UN, we collected synovial tissue from OA patients and rinsed it with DIO‐labeled MI@UN. Frozen sections were then made to verify the targeting effect. CD68 labels synovial macrophages and DIO labels MI@UN, showing almost overlapping DIO and CD68 (Figure S6I, Supporting Information). We also collected OA cartilage tissue from OA patients and rinsed it with the same method. Only slight green fluorescence was seen on the cartilage surface, demonstrating MI@UN's targeting of synovial macrophages in vitro (Figure S6G, Supporting Information). We then performed cytotoxicity assays on the components of MI@UN in both macrophages and chondrocytes. The results showed that the use of MM or IL‐10 alone had little effect on the cells. However, cell viability significantly decreased when the NO‐prednisolone concentration exceeded 10 µM. When the UIO‐66‐NH_2_ concentration was above 80 µg mL^−1^, the cell viability also significantly decreased. The toxicity of UIO‐66‐NH_2_ was significantly reduced because of the coating of the MM. At a concentration of 120 µg mL^−1^, the viability of the MI@UN‐treated cells was similar to that of the 80 µg mL^−1^ UIO‐66‐NH_2_‐treated cells (Figure 6F). To verify the biosafety of MI@UN, we divided mice into two groups: a control group with no intervention and a MI@UN group that received weekly intra‐articular injections of MI@UN for a month. Ocular blood was collected for routine blood tests and biochemistry. Results showed MI@UN had almost no effect on the mice's indices, confirming its in vivo biosafety (Figure S6E, Supporting Information). We also measured the particle sizes of UIO‐66‐NH_2_ and MI@UN, with UIO‐66‐NH_2_ diameters concentrated at ≈100 nm and MI@UN diameters at ≈110 nm (Figure 6G). The absence of IL‐10Rα reduces M2 macrophage expression, while MI@UN can enhance M2 macrophage expression (Figure S7A, Supporting Information). To verify the protective effect of MI@UN, macrophages and chondrocytes were co‐cultured with it. Results showed that MI@UN could relieve LPS‐induced polarization and promote M2 polarization of macrophages (Figure S7B, Supporting Information), and protect chondrocytes from erastin's effects (Figure S7C,D, Supporting Information). After confirming the protective effects of MI@UN on macrophages and chondrocytes, we assessed its impact on macrophage glycolysis and chondrocyte ferroptosis. Our WB analysis revealed that MI@UN significantly curtailed hypoxia‐induced excessive glycolysis in macrophages and markedly influenced the IL‐10Rα/glycolysis axis (Figure S7E,F, Supporting Information). Consistently, when we co‐cultured chondrocytes with macrophage supernatants, we found that inhibiting excessive macrophage glycolysis markedly alleviated chondrocyte ferroptosis, echoing our prior findings (Figure S7G,H, Supporting Information). In summary, by coating with a MM, we successfully encapsulated the modified UIO‐66‐NH_2_, targeted the delivery of IL‐10 and NO‐prednisolone. MI@UN not only protected macrophages and chondrocytes but also influenced the IL‐10/IL‐10Rα/glycolysis axis, meeting our design objectives.

### 2.7 Therapeutic Effects of MI@UN in Animal Models

**Figure 7:**
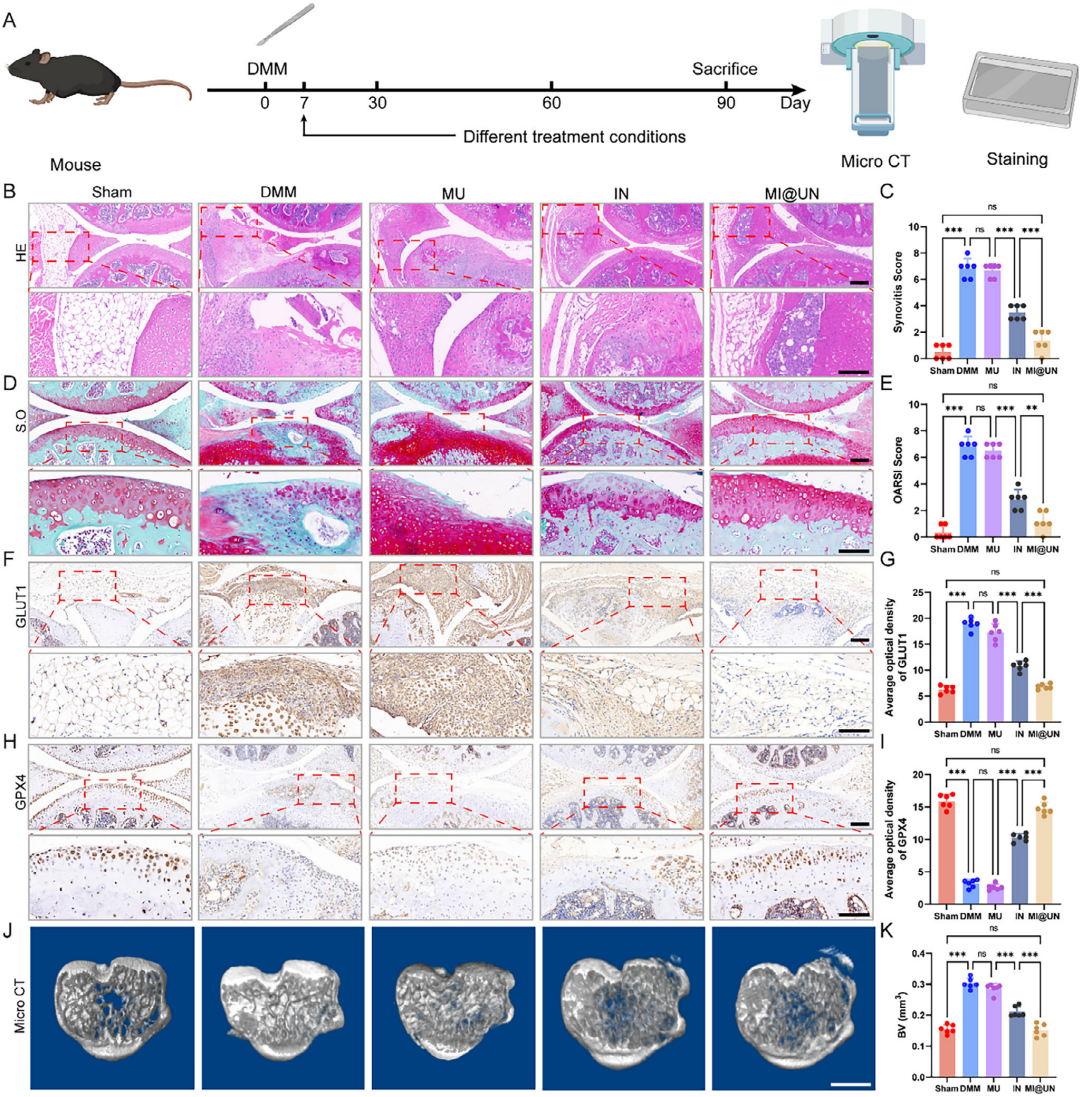
Effects of MI@UN on the synovium and cartilage in OA. A) Schematic of the process for inducing DMM in mice, treatment, sampling, and subsequent experiments. B) HE staining of synovial tissue from the groups of mice. Scale bar: 100 µm. C) Quantification of the synovitis scores of the synovial tissue. D) S.O. staining of cartilage tissue. Scale bar: 100 µm. E) Quantification of the OARSI. F,G) Immunohistochemical staining of GLUT1 in synovial tissue and quantification of positive areas. Scale bar: 100 µm. H,I) Immunohistochemical staining of GPX4 in cartilage tissue and quantification of positive areas. Scale bar: 100 µm. J,K) Top Micro‐CT view of the subchondral bone and quantitative analysis of BV in the sham and DMM groups of mice (*n* = 6). Scale bar: 1 mm. ^*^ *p* < 0.05, ^**^ *p* < 0.01, ^***^ *p* < 0.001, ^****^ *p* < 0.001, ns indicates not significant.

For histological studies, we induced DMM in mice by injuring the meniscus and randomly divided them into groups to receive different treatments. Ninety days post‐treatment, the joints of the mice were harvested to further assess the impact of the MI@UN functionalized biomimetic nanoparticles on OA (**Figure** **7**A). To verify the retention time of MI@UN in the mouse joint cavity, we labeled IL‐10 and NO‐prednisolone with CY5.5 and observed the retention time directly after injection via in vivo imaging. Imaging revealed that without the MM and UIO‐66‐NH_2_, IN was maintained for only 2 days, whereas MI@UN achieved a sustained release time of up to 14 days, significantly maintaining the drug concentration and extending the therapeutic timeframe (Figure S8A, Supporting Information). We also encapsulated IL‐10 alone with the MM and observed an extension of the IL‐10 retention time, exploring its potential as a standalone therapy (Figure S8B, Supporting Information). HE staining of all harvested joints revealed synovial hyperplasia. The synovial tissue structure of the sham group was clear, whereas that of the DMM group exhibited dense and disordered cell proliferation. Compared with the sham group, the MU group had little effect, with synovial hyperplasia still present, although IN had an initial therapeutic effect, reducing the density of the synovial tissue. However, the combination of IL‐10 and NO‐prednisolone without UIO‐66‐NH_2_ and MM did not achieve the optimal therapeutic effect, although some improvement in the synovium was observed. Owing to the modification of UIO‐66‐NH_2_ and encapsulation with the cell membrane, MI@UN injection resulted in the best therapeutic effect, with the synovial structure clearly resembling that of the sham group and the cartilage showing no depressions or cracks, with a relatively smooth surface and normal cartilage thickness (Figure 7B). Treatment with MI@UN significantly reduced the synovitis score (Figure 7C). S.O. staining revealed that the normal cartilage layer exhibited uniform staining with clear layers, whereas the DMM group presented severe cartilage wear and an irregular cartilage surface with uneven staining. The MU group still presented severe cartilage wear and abnormal cartilage distribution, although the IN group presented some repair effects. However, the therapeutic effect of MI@UN was more significant, with a marked reduction in cartilage erosion and uneven cartilage surfaces and cracks. IN treatment reduced the OA score but lacked targeting and sustained‐release functions, and its therapeutic effect in both spatial and temporal terms was significantly inferior to that of MI@UN (Figure 7D,E). Similarly, we performed IHC staining of the synovium to detect GLUT1 expression. We found that the DMM and MU groups with synovial hyperplasia both presented strong GLUT1 expression, whereas IN, owing to its short half‐life, did not achieve the optimal therapeutic effect. The addition of MI@UN significantly alleviated expression of GLUT1, with a therapeutic effect far exceeding that of direct IN injection. This finding is consistent with our predicted results, as the protection of the MM and sustained release of UIO‐66‐NH_2_ significantly promoted the therapeutic effect on the synovium, with synovial macrophages phagocytosing MI@UN successfully alleviating excessive synovial glycolysis (Figure 7F,G). Moreover, we performed IHC staining of the cartilage to detect ferroptosis. We found that the expression of GPX4 in the DMM and MU groups was very low, with almost no expression in areas of severe cartilage wear, whereas the expression in the MI@UN group was almost identical to that in the sham surgery group (Figure 7H,I). We also scanned the subchondral bone of the mice and found that the subchondral bone structure of the sham group was porous, while the DMM group exhibited substantial cartilage hardening, and the porous structure disappeared. Quantitative analysis revealed that the BV in the DMM group was significantly increased. IN treatment had certain therapeutic effects, with some improvement in the dense structure, but after treatment with MI@UN, and osteophyte was reduced, similar to that in the sham surgery group (Figure 7J,K). Quantitative analysis of BV, bone density, Tb.N, and bone volume fraction further revealed that the MI@UN group approached the level of the sham surgery group, indicating that MI@UN had a therapeutic effect on the subchondral bone (Figure S8C,D, Supporting Information). To verify the activity of IL‐10R, we performed immunohistochemical staining for IL‐10R in sections from the sham, DMM, and MI@UN groups and found that the expression of IL‐10R increased with MI@UN treatment (Figure S8E,F, Supporting Information). We also harvested the main organs of the mice injected with MI@UN and detected no toxic accumulation in the main organs, and reproductive function was not affected (Figure S8G, Supporting Information). In our study, the therapeutic effect of MI@UN was compared to that of clinically standardized drugs. We found that MI@UN achieved a therapeutic effect comparable to that of clinically standardized drugs. In both treatment groups, the synovial structure was clear (Figure S9A,B, Supporting Information) and the cartilage surface smooth (Figure S9C,D, Supporting Information), with similar GLUT1 expression (Figure S9E, Supporting Information). However, after MI@UN treatment, GPX4 expression was higher (Figure S9F, Supporting Information), possibly due to IL‐10 supplementation. MI@UN provided more GPX4, contributing to chondrocyte defense against ferroptosis. We provided gait analysis and gait assessment analysis of mice to judge the therapeutic effect of MI@UN on DMM mice. In OA mice, toe spread was smaller, and step width and length were reduced. After MI@UN treatment, these parameters normalized, and the gait score was similar to the control group (Figure S10A,B, Supporting Information). In summary, these data collectively emphasize the potential of MI@UN as an effective treatment for OA. These findings support the clinical application prospects of MI@UN in the treatment of OA, as it almost completely restores the parameters of a normal joint and has a therapeutic effect on subchondral bone.

## 3 Discussion

**Figure 8:**
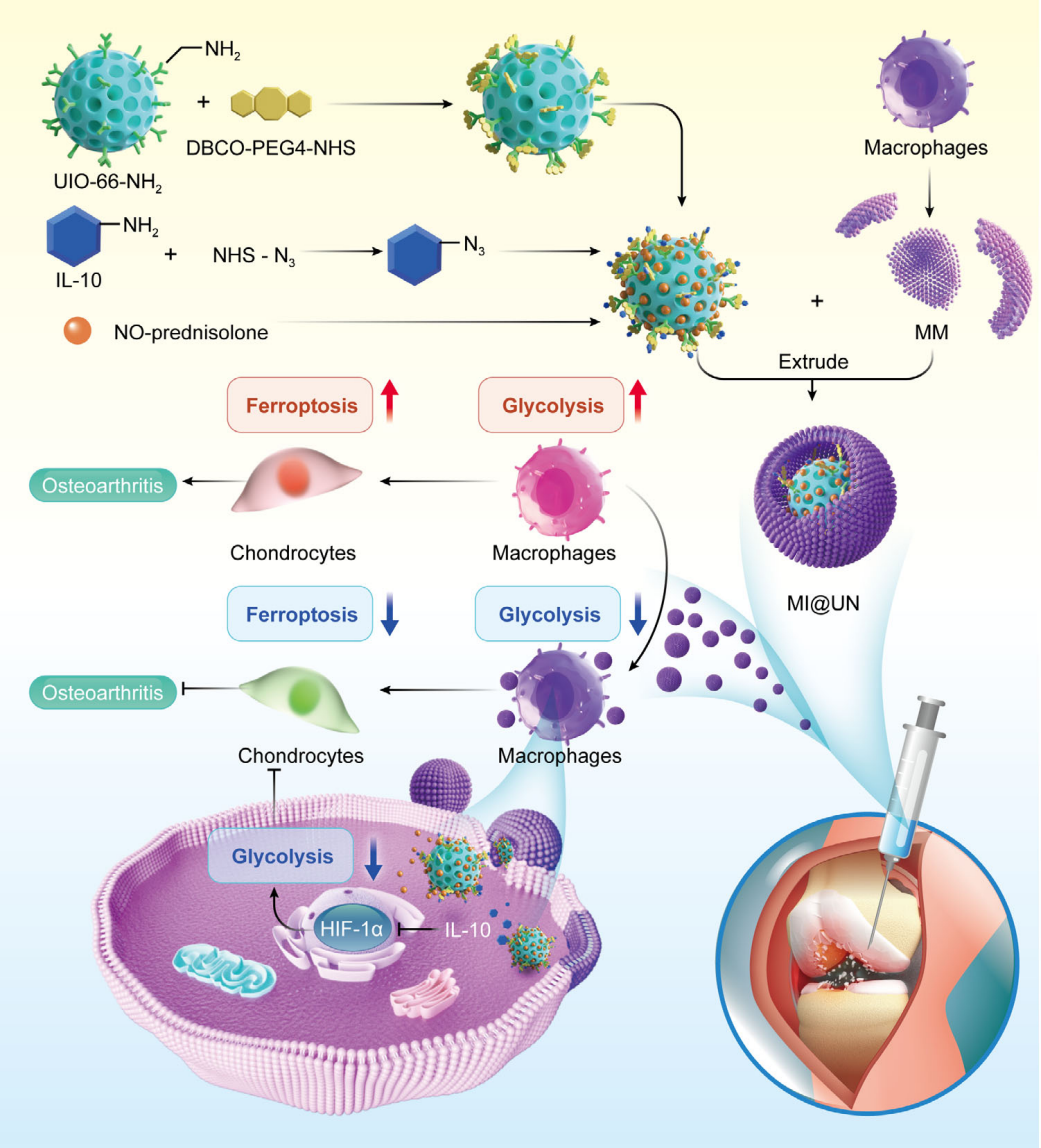
Schematic diagram. The MOF material UIO‐66‐NH_2_ is functionalized with DBCO groups on its surface, encapsulating NO‐prednisolone and conjugating it with azide‐modified IL‐10. Finally, the complex is extruded with MM to form biomimetic functional nanoparticles. Upon intra‐articular injection, MI@UN, enveloped by the MM, specifically targets synovial macrophages, is phagocytosed, and accumulates in the synovial region. Ultimately, MI@UN alleviates the glycolysis of synovial macrophages and the ferroptosis of chondrocytes, effectively treating OA.

Using myeloid‐specific IL‐10Rα knockout mice, we established for the first time the regulatory relationship between glycolysis in synovial macrophages and ferroptosis in chondrocytes and explored the potential downstream pathways of the IL‐10/IL‐10Rα axis. Our findings reveal that IL‐10Rα can modulate excessive glycolysis in macrophages through HIF‐1α, thereby alleviating ferroptosis in chondrocytes and attenuating the progression of OA. Furthermore, we engineered a functionalized biomimetic nanoparticle, MI@UN, which extends the retention time of IL‐10, maintains local drug concentrations, and addresses the issue of nontargetability (**Figure** **8**).

OA, the most common chronic musculoskeletal disorder, is marked by the deterioration and depletion of cartilage, impacting all joint structures, such as the synovium and subchondral bone.^[^ ^29^ ^]^ The evidence indicates that synovitis is linked to the onset and advancement of OA.^[^ ^30^ ^]^ Initially considered a manifestation of advanced OA,^[^ ^31^ ^]^ synovitis was later found to be present in 83% of OA patients at early stages,^[^ ^5^ ^]^ often preceding structural changes in bone and cartilage.^[^ ^4c^ ^]^ This finding has increased the focus on synovitis as a key factor in OA. The survival of chondrocytes is crucial for the amelioration of OA. Ferroptosis in chondrocytes is common in OA^[^ ^32^ ^]^ and accelerates the progression of this disease.^[^ ^33^ ^]^ Given the anatomical proximity of the synovium and cartilage within the joint cavity and their indirect contact mediated by synovial fluid, we investigated the crosstalk between synovial macrophages and chondrocytes. For the first time, we identified the regulatory relationship between macrophage glycolysis and chondrocyte ferroptosis. We observed excessive and robust energy metabolism in both OA patients and mice with OA, which we hypothesize is due to the inflammatory microenvironment created by OA. This environment polarizes macrophages, affecting metabolic processes and leading to the production of cytokines that impact chondrocyte survival. Additionally, there exists a substantial variation in the levels of expression of the classic anti‐inflammatory factor receptor IL‐10Rα between the normal synovium and osteoarthritic tissue, providing a rationale for exploring the cellular crosstalk and mechanism of IL‐10/IL‐10Rα.

To elucidate the mechanism of IL‐10/IL‐10Rα, we isolated primary synovial macrophages from DMM‐treated, 4‐month‐old IL‐10Rα^Lysm^ mice and their littermate WT mice and performed RNA sequencing to identify downstream pathways and proteins. Among the 306 differentially expressed genes associated with altered energy metabolism, HIF‐1α was the most significant. HIF‐1α, a nuclear transcription factor often expressed under hypoxic conditions,^[^ ^34^ ^]^ regulates the expression of glycolytic pathway genes, thus facilitating the cellular energy supply and survival under low‐oxygen conditions.^[^ ^35^ ^]^ Therefore, we investigated the relationships among IL‐10Rα, HIF‐1α, and glycolysis. We silenced IL‐10Rα in macrophages, assessed the glycolytic markers HK1, LDHA, and GLUT1 and found that IL‐10Rα can alleviate excessive glycolysis in synovial macrophages. Furthermore, we added LW6 (a HIF‐1α inhibitor) to macrophages and observed the suppression of glycolysis. These findings suggest that the IL‐10/IL‐10Rα axis may regulate the glycolytic process in macrophages through HIF‐1α. To explore the impact of synovial macrophages on chondrocytes, we cocultured chondrocytes with CM from macrophages and assessed ferroptosis in chondrocytes. We found that macrophage‐CM exerts a regulatory effect on chondrocyte ferroptosis, possibly through lactic acid secreted by macrophages that influence chondrocyte ferroptosis. On this basis, we conclude that the IL‐10/IL‐10Rα axis, through HIF‐1α, regulates the glycolytic process in synovial macrophages, which in turn affects chondrocyte ferroptosis.

IL‐10 is a candidate for a range of diseases, but its short half‐life and off‐target effects severely hinder its clinical translation.^[^ ^36^ ^]^ Functionalized biomimetic nanoparticles coated with cells have shown good therapeutic potential,^[^ ^37^ ^]^ inheriting parental cell characteristics such as specific targeting^[^ ^27b^ ^]^ and drug delivery.^[^ ^38^ ^]^ Therefore, we developed a functionalized biomimetic nanoparticle, MI@UN, to achieve targeted delivery, increase therapeutic efficacy, and maintain local drug concentrations for an extended period. In our study, the MM encapsulated the modified UIO‐66‐NH_2_, and due to the MM coating, MI@UN was actively phagocytosed by the macrophages. IL‐10 was effectively conjugated on the surface of the MOF material UIO‐66‐NH_2_ and released in large amounts shortly after injection. To increase the efficacy of IL‐10, we loaded the IL‐10 agonist NO‐prednisolone into UIO‐66‐NH_2_. NO‐prednisolone encapsulated within UIO‐66‐NH_2_ achieved long‐term slow release, maintaining local drug concentrations. Compared with the IN group, MI@UN had an effect that lasted up to 14 days and was biocompatible, with no impact on vital organs after degradation. While direct injection of IN had some therapeutic effects, it was not satisfactory, and the improvements in the subchondral bone and synovium were not significant. The occurrence of active phagocytosis by macrophages exceeded our expectations, providing a solid foundation for the therapeutic potential of MI@UN.

In conclusion, we identified differences in IL‐10Rα in OA and revealed that the IL‐10/IL‐10Rα axis functions through the regulation of HIF‐1α. We also elucidated the process by which IL‐10Rα modulates macrophage glycolysis via HIF‐1α and discovered the link and regulatory relationship between synovial macrophage glycolysis and chondrocyte ferroptosis via coculture methods. To achieve a better therapeutic effect, we developed a MM‐coated functionalized biomimetic nanoparticle, MI@UN, which serves as a targeted delivery system and provides sustained release of IL‐10 and NO‐prednisolone. This study provides new insights into the novel therapeutic target IL‐10Rα for OA and offers potential for the clinical translation of MM‐coated MOF materials.

## 4 Experimental Section

### Ethics Statement

All surgical procedures, medication administrations, and postoperative care for the animals were conducted in strict accordance with the guidelines of the Animal Center at the First Affiliated Hospital of USTC, Anhui Provincial Hospital (2023‐N(A)‐106).

### Human Samples

Tissues from healthy synovial and articular cartilage were procured from individuals who underwent amputation without a prior diagnosis of arthritis. Conversely, tissues affected by osteoarthritis were gathered during total knee replacement procedures. Patients with OA due to articular cartilage degeneration were included, and those with severe diseases such as malignant tumors or diabetes within the past 5 years were excluded. Human synovial tissues were subjected to HE staining, IF, and immunohistochemistry (IHC) staining. Human specimens were collected at the First Affiliated Hospital of USTC, also referred to as Anhui Provincial Hospital. Written informed consent was obtained from all participants, allowing the use of their clinical data for research. The study protocol was approved by the Institutional Review Board of the First Affiliated Hospital of USTC, Anhui Provincial Hospital (2023‐KY‐371).

### Preparation and Characterization of Nanoparticles

Pluronic F127 (50 mg) was dissolved in 3 mL of deionized water, followed by the addition of 0.4 mL of acetic acid (AA) and 150 mg of NaClO_4_·H_2_O and thorough mixing until a homogeneous solution formed. Then, 115.6 mg of ZrO (NO_3_)_2_·2H_2_O and 50 mg of BDC‐NH_2_ were added to the aforementioned mixture. After magnetic stirring at 40 °C for 12 h, the mixture was centrifuged at 10000 rpm to obtain the precipitate. The precipitate was washed once with deionized water and then centrifugally separated again. The product was resuspended in anhydrous ethanol and then preserved. One milligram per milliliter of UIO‐66‐NH_2_ was mixed with 1.25 mg dissolved in 50 µL of NO‐prednisolone at room temperature (RT) and magnetically stirred for 24 h, followed by centrifugation to obtain the precipitate. One milligram per milliliter of UIO‐66‐NH_2_ loaded with NO‐prednisolone was reacted with 10 mmol L^−1^ DBCO‐PEG_4_‐NHS in PBS at RT. After a 4‐h reaction, the mixture was centrifuged at 12 000 × g for 10 min in an ultrafiltration tube and washed three times with PBS to remove any unreacted DBCO‐PEG_4_‐NHS. Subsequently, azide‐modified IL‐10 was synthesized through the reaction between NHS‐N_3_ (6 mL of 64 ng mL^−1^) and IL‐10 (5 µg). Then, azide‐labeled IL‐10 was added to NO‐UIO‐DBCO and magnetically stirred at 4 °C for 4 h, followed by centrifugation in an ultrafiltration tube to obtain purified I@UN.

The nanoparticle preparation was proceeded with by culturing RAW264.7 cells. When the cell density reached 80%, the macrophages were collected and resuspended in Tris‐magnesium salt buffer (TM buffer) (1 mm MgCl_2_ and 10 mm Tris, with deionized water adjusted to pH 7.4). After resuspension, the cells were lysed via an ultrasonic homogenizer on ice. Following lysis, the cell homogenate was mixed with a 1 m sucrose solution to achieve a final concentration of 0.25 m sucrose. The resulting product was centrifugally separated to purify the cell membrane. The extracted MM was quantified via the BCA protein assay to determine the protein concentration.

Finally, the MM obtained from the previous step was mixed with I@UN (0.05 mg of MM and 0.025 mg mL^−1^ of I@UN, 1:5 ratio) and extruded via a miniextruder through a polycarbonate filter 20 times to obtain MI@UM. For evaluation of the encapsulation of NO‐prednisolone and the successful conjugation of IL‐10, N_3_‐CY5.5‐NHS‐labeled IL‐10, CY3‐labeled NO‐prednisolone, and the MM stained with DIO were used, with DAPI marking the nuclei of the macrophages. These four fluorescently labeled MI@UN nanoparticles were visualized via confocal laser scanning microscopy (CLSM; Zeiss, Germany).

### Transmission Electron Microscopy (TEM)

Ten microliters of the prepared UIO‐66‐NH_2_, MM, and MI@UN suspensions were dispensed onto a Parafilm sheet. The copper grid was placed with the carbon side up onto the droplet and allowed to adsorb for 10–15 min. A pipette was then used to apply 10 µL of a 2% phosphotungstic acid solution onto the Parafilm. The grid, which was then coated with the sample, was inverted onto the staining solution and allowed to sit for 3–5 min to stain. The excess liquid was removed with filter paper, and the mixture was air dried under an incandescent lamp. The samples were observed and photographed via TEM.

### Materials

Fetal bovine serum (Cat#35‐105‐CV) purchased from Corning. DMEM (Cat#RG‐CE‐2) purchased from Ketu Biotech, trypsin (Cat#25200056) was from Gibco, erastin (Cat#HY‐15763) was from MCE, and all antibodies, including HIF‐1α, IL‐10Rα, GLUT1, LDHA, HK1, ACSL4, and β‐actin, were obtained from Proteintech, GPX4 was obtained from abcam. RIPA buffer, a BCA protein assay kit, and DIO were obtained from Beyotime, and the Cell Counting Kit‐8 (CCK‐8) was purchased from Yeasen. The IL‐10 cytokine, NO‐prednisolone, CY5.5, CY3, and DBCO‐PEG_4_‐NHS were procured from MCE; AA, NaClO_4_·H_2_O, ZrO (NO_3_)_2_·2H_2_O, and BDC‐NH_2_ were obtained from Aladdin; Pluronic F127 was obtained from Sigma; and NHS‐N_3_ was obtained from Meryer.

### Mice

IL‐10Rα myeloid‐specific knockout mice were generously provided by Professor Bofeng Li.

Adult male C57 mice (25–30 g) were purchased from the Anhui Provincial Hospital Animal Center (Anhui, Hefei). All the mice were housed in standard experimental cages with a 12‐h light‒dark cycle and had free access to water and standard chow. All operators participated in and passed the animal laboratory training. In brief, DMM involves the anesthesia of mice with isoflurane, the removal of excess hair, the opening of the joint capsule, and damage to the meniscus to induce the wear of articular cartilage, leading to the onset of OA. This study utilized a model of DMM‐affected mice, with no mortality within the first 7 days post‐surgery. After 7 days, the mice were randomly divided into the sham, DMM, MM, IN, and MI@UM groups for subsequent experiments. Sham group: Mice underwent intra‐articular opening without additional treatment, then joint cavity suturing. This group served as a normal control. DMM group: After meniscal injury, only joint cavity suturing was done, no treatment. This group provided a natural OA progression model for comparison with treatment groups. MM group: After meniscal injury, intra articular injections of purified, fragmented macrophage membranes were administered weekly. Each injection was 50 µL (0.05 mg), to study their effects on meniscal injury and OA development. IN group: After meniscal injury, weekly intra – articular injections of a mixture of IL‐10 (100 ng) and NO‐prednisolone (5 µg) were performed. Each injection was 50 µL, to evaluate its efficacy and safety in OA treatment. MI@UN group: One week post‐meniscal injury, weekly intra‐articular injections of MI@UN were performed. Each injection was 50 µL, containing 10 µg of MI@UN, to explore its effects on meniscal injury and OA treatment. Treatment lasted 12 weeks. Aseptic technique was used for injections to prevent complications like infection. Mice health was closely monitored.

### In Vivo Retention of Fluorescent Labeling (Live Imaging)

Following the fluorescent tagging, the substances were administered into the mice's joint spaces to ascertain the duration of retention. Specifically, CY5.5 was used to label MI@UM according to the manufacturer's instructions. Images were acquired at intervals after injection. Each time point was represented by a set of three separate images. The region of interest (ROI) feature in Living Image 4.3.1 software was utilized to quantify the fluorescence intensity at each joint site.

### Micro‐CT

The murine knee joints were excised and immersed in paraformaldehyde for fixation. All knee joints obtained were subjected to Micro‐CT (Skyscan 1176, Belgium) scans. The settings for resolution were configured to 10 µm, with an acceleration voltage of 70 kV and a probe current of 114 µA. The (ROI) was delineated to encompass the full tibial plateau and subchondral bone, then analyzed metrics including bone volume (BV), the ratio of bone volume to total tissue volume (BV/TV), and trabecular thickness (Tb.Th).

### Histological Observation

Following decalcification with a 10% EDTA solution, the specimens were embedded in paraffin. Sections of the knee joint's medial compartment, cut to a thickness of 4 µm, the samples were subjected to Hematoxylin and Eosin (HE) and Safranin O (S.O) staining. Following this, the Osteoarthritis Research Society International (OARSI) scoring system along with the synovitis grading criteria were applied to assess the outcomes. Fluorescence and immunohistochemical staining were performed with antibodies against GLUT1 and GPX4, and images were captured via light microscopy.

### Cell Culture

RAW264.7 and ATDC5 were obtained from the Cell Bank of the Chinese Academy of Sciences in Shanghai, China. Both cell types were cultured in Dulbecco's Modified Eagle Medium (DMEM) supplemented with 10% fetal bovine serum and maintained at 37 °C under a humidified atmosphere containing either 5% CO_2_ or 1% O_2_. While RAW264.7 cells were harvested without the use of trypsin, ATDC5 cells required trypsin treatment. Once the cells reached ≈80–90% confluence, they were passaged at a 1:3 dilution ratio every two weeks. The expanded cells were then used for subsequent experimental procedures.

### Cytotoxicity

A CCK‐8 assay was used to assess cell viability. RAW264.7 and ATDC5 cells were seeded into 96‐well plates at a density of 5000 cells per well. After 12 h, the MM (final concentrations of 2, 4, 6, 8, 10, 12, 14, 20, 40 µg mL^−1^), NO‐prednisolone (final concentrations of 2, 4, 6, 8, 10, 12, 14, 20, 40 µM), IL‐10 (final concentrations of 2, 4, 6, 8, 10, 12, 14, 20, 40 ng mL^−1^), and UIO‐66‐NH_2_ (final concentrations of 20, 30, 40, 50, 60, 70, 80, 100, 120 µg mL^−1^) were added to each well in fresh culture medium. The cells were incubated for 24 h at 37 °C in a humidified incubator with 5% CO_2_. CCK‐8 reagent was added to each well, and the cells were incubated for an additional 2 h at 37 °C. The absorbance at 450 nm was measured via a microplate reader. Cell viability was calculated with the control group set at 100%, and each well was performed in six replicates.

### Western Blot (WB)

Protein extraction from cells and tissues was performed using a radioimmunoprecipitation assay (RIPA) buffer supplemented with 10% phosphatase inhibitors and 1% phenylmethylsulfonyl fluoride (PMSF). The cells were plated in 6‐well plates and treated accordingly when they reached 80% confluence. After 24 h, RIPA lysis buffer supplemented with 10% phosphatase inhibitors and 1% PMSF was used to extract total protein. Samples were prepared for analysis by boiling to denature proteins, followed by centrifugation to isolate the supernatant. Total protein content was quantified using the bicinchoninic acid (BCA) protein assay kit and then normalized. Following this, the samples were subjected to sodium dodecyl sulfate‐polyacrylamide gel electrophoresis (SDS‐PAGE) and transferred to a nitrocellulose membrane. The membrane was blocked with skim milk for 30 min before incubation with various primary antibodies at different dilutions overnight at 4 °C. Subsequently, the membranes underwent three washes with Tris Buffered Saline with Tween 20 (TBST), followed by incubation with the appropriate secondary antibodies for 1 h. Chemiluminescent detection was carried out using a film developer, with β‐actin as the internal control. Band intensities were quantified using ImageJ software to determine the relative protein expression levels. The antibodies used for protein detection included HIF‐1α, IL‐10Rα, GLUT1, LDHA, HK1, ACSL4, GPX4, and β‐actin. Each experiment was performed in triplicate to ensure reproducibility.

### Primary Cells

IL‐10Rα^Lysm^ mice and their wild‐type (WT) littermates were euthanized following DMM treatment. The knee joints of the mice were removed, the synovial tissue was dissected, and the collected tissue was immediately placed in cold PBS buffer. The synovial tissue was diced into 1–2 mm^3^ fragments and immersed in a solution comprised of digestive enzymes. This mixture was incubated at 37 °C in a water bath for a duration between 30 and 60 min, with occasional gentle agitation to facilitate the digestion process. Post‐digestion, the solution was strained through a 70 µm mesh cell strainer, and the resulting cell suspension was transferred to conical tube. The suspension was then centrifuged at 1500 rpm at 4 °C for 5 min, after which the supernatant was decanted, leaving the cell pellet to be resuspended in chilled PBS. The processed solution was strained into a precooled centrifuge tube and spun at 1500 rpm at 4 °C for 5 min. Upon completion of the centrifugation, the supernatant was aspirated, followed by the addition of 5 mL of PBS. The tube was then centrifuged again at the same speed and temperature for another 5 min. The cells from the single‐cell suspension were enumerated after trypan blue staining, and their concentration was adjusted to a density of 1 × 10⁷ cells mL^−1^. Subsequently, F4/80 antibody‐conjugated magnetic beads were introduced, following the manufacturer's guidelines. Then, 10 µL of magnetic beads was added to 1 mL of single‐cell suspension, gently mixed, and incubated at 4 °C for 15–30 min to ensure adequate binding of the magnetic beads to the F4/80 antigen on the macrophage surface. After incubation, the cell suspension was transferred to a magnetic separation column. The F4/80‐positive cells were retained via magnetic force, the column was washed three times with cold PBS to remove other unbound cells, and finally, the F4/80‐positive macrophages were eluted and collected.

### RNA Sequencing

Primary cells were derived from the synovium of IL‐10Rα^Lysm^ and WT mice subjected to DMM and subsequently were divided into groups. The cells were divided into IL‐10Rα^Lysm^ and WT groups, with each group containing three replicate samples, to increase the accuracy of the data. Total RNA was isolated and its purity was determined. RNA integrity was determined using the Agilent 2100 Bioanalyzer, which is manufactured by Agilent Technologies in the USA. Thereafter, the VAHTS Universal V6 RNA‐seq Library Prep Kit was utilized for library preparation, adhering to the manufacturer's protocol. RNA purification, reverse transcription, library construction, and sequencing were conducted at Hangzhou Cosmos Wisdom Biotechnology Co., Ltd. (Hangzhou, China).

### Fluorescence

The cells were rinsed with chilled PBS, followed by fixation using 4% paraformaldehyde on ice for a duration of 15 min, and subsequently rinsed with PBS three times. The samples were permeabilized with Triton X‐100 in immunostaining permeabilization solution at RT for 10 min, followed by blocking with goat serum blocking solution (Beyotime) for 1 h. Post‐blocking, the cells were treated with specific primary antibodies for an extended period overnight. On the following day, the primary antibodies were reapplied. After washing, the samples were incubated with their corresponding secondary antibodies in the dark at room temperature for 1 h. The cells were then stained with 4,6‐diamidino‐2‐phenylindole (DAPI) for 5 min to visualize the nuclei. An antifade mounting medium was used to mount the cover slips. The cells were examined and imaged using a confocal microscope.

### IHC Analysis

The tibial and femoral tissues were carefully dissected, fixed in a 10% formalin solution, decalcified using a 10% EDTA solution for a week, and then dehydrated through a sequential treatment with alcohols of increasing concentration. These tissues were then embedded in paraffin, and 4 µm serial sections were prepared. For each mouse, three sections, all originating from the femoral medial condyle, were examined. Endogenous peroxidase activity was quenched with a 3% H_2_O_2_ solution, and nonspecific binding was blocked with 5% BSA. The sections were incubated with primary antibodies at 4 °C overnight. Following this, the sections were treated with the appropriate horseradish peroxidase‐conjugated secondary antibodies for further incubation. The sections were then stained with hematoxylin and eosin (HE) as per the manufacturer's protocol.

### Collection of Human Specimens

Synovial and joint platform tissues were collected from OA patients who required amputation and total knee replacement with patient consent. Synovial and cartilage tissues were separately collected, embedded in dehydrating paraffin, sectioned and stained. The staining methods included HE and IF. All human samples were obtained from the First Affiliated Hospital of USTC, Anhui Provincial Hospital. This study was approved by the Ethics Committee of the First Affiliated Hospital of USTC, Anhui Provincial Hospital.

### Statistical Analysis

Data are presented as mean values ± standard deviations, obtained from at least three independent experimental trials. Statistical analyses were performed using GraphPad Prism version 9 from GraphPad Software, Inc., based in California, USA. T‐tests were used for pairwise comparisons, while analysis of variance (ANOVA) followed by post hoc paired t‐tests were employed for multiple group comparisons. A P value of less than 0.05 was considered statistically significant.

## Conflict of Interest

The authors declare no conflict of interest.

## Author Contributions

W.L., Y.L., and M.W. contributed equally to this work and are the first co‐authors. W.L. designed the experiment and wrote the manuscript. Y.L. completed the collection and sorting of human samples. M.W. was involved in the main experiments. Z.Y., Z.G., Z.L., L.Y., and Y.L., completed the analysis of the data. All authors contributed to drafting the article or discussing important sections, and all gave their consent for final publication. T.H. provided constructive guidance for the experiment and revised the experimental framework; L.B. participated in the guidance of the experiment and provided valuable mice; H.W. guided the experiment, revised the manuscript, and provided financial assistance.

## Supporting information



Supporting Information

## Data Availability

The data that support the findings of this study are available from the corresponding author upon reasonable request.
