# Supplementary material for: Functionalized Biomimetic Nanoparticles Targeting the IL‐10/IL‐10Rα/Glycolytic Axis in Synovial Macrophages Alleviate Cartilage Degeneration in Osteoarthritis
Source: Adv Sci (Weinh). 2025 May 2;12(27):2504768. doi: 10.1002/advs.202504768 (PMC12279240; doi:10.1002/advs.202504768)
Supplement: Supplementary file 1 — Supporting Information [file ADVS-12-2504768-s001.docx]

Supplementary Materials for

**Functionalized Biomimetic Nanoparticles Targeting the IL-10/IL-10Rα/Glycolytic Axis in Synovial Macrophages Alleviate Cartilage Degeneration in Osteoarthritis**

Wenwei Li^†^, Yang Liu^†^, Ming Wei^†^, Zhichao Yang, Zhaoyu Li, Zezhong Guo, Liang Yan, Yang Lu, Hao Tang^*^, Bofeng Li^*^, Wei Huang^*^

W. Li, Y. Liu, M. Wei, Z. Yang, Z. Li, Z. Guo, L. Yan, W. Huang

Department of Orthopedics, Centre for Leading Medicine and Advanced Technologies of IHM, The First Affiliated Hospital of USTC, Division of Life Sciences and Medicine, University of Science and Technology of China

Hefei, Anhui, 230001, China

E-mail: zgkdhwei@ustc.edu.cn

W. Li, Z. Guo

School of Medicine, Anhui University of Science and Technology

Huainan, Anhui 232000, China

H. Tang, Y. Lu

Department of Orthopedics, The First Affiliated Hospital of Anhui Medical University

Hefei, Anhui, 230001, China

E-mail: 19908866@qq.com

B. Li

Department of Medical Oncology, The First Affiliated Hospital of USTC, Division of Life Sciences and Medicine, University of Science and Technology of China Hefei, Anhui, 230001, China

Institute of Health and Medicine, Hefei Comprehensive National Science Center

Hefei, Anhui, 230001, China

E-mail: [libf@ustc.edu.cn](mailto:libf@ustc.edu.cn).

This PDF file includes the following:

Fig. S1 to S10

Tables S1 to S5


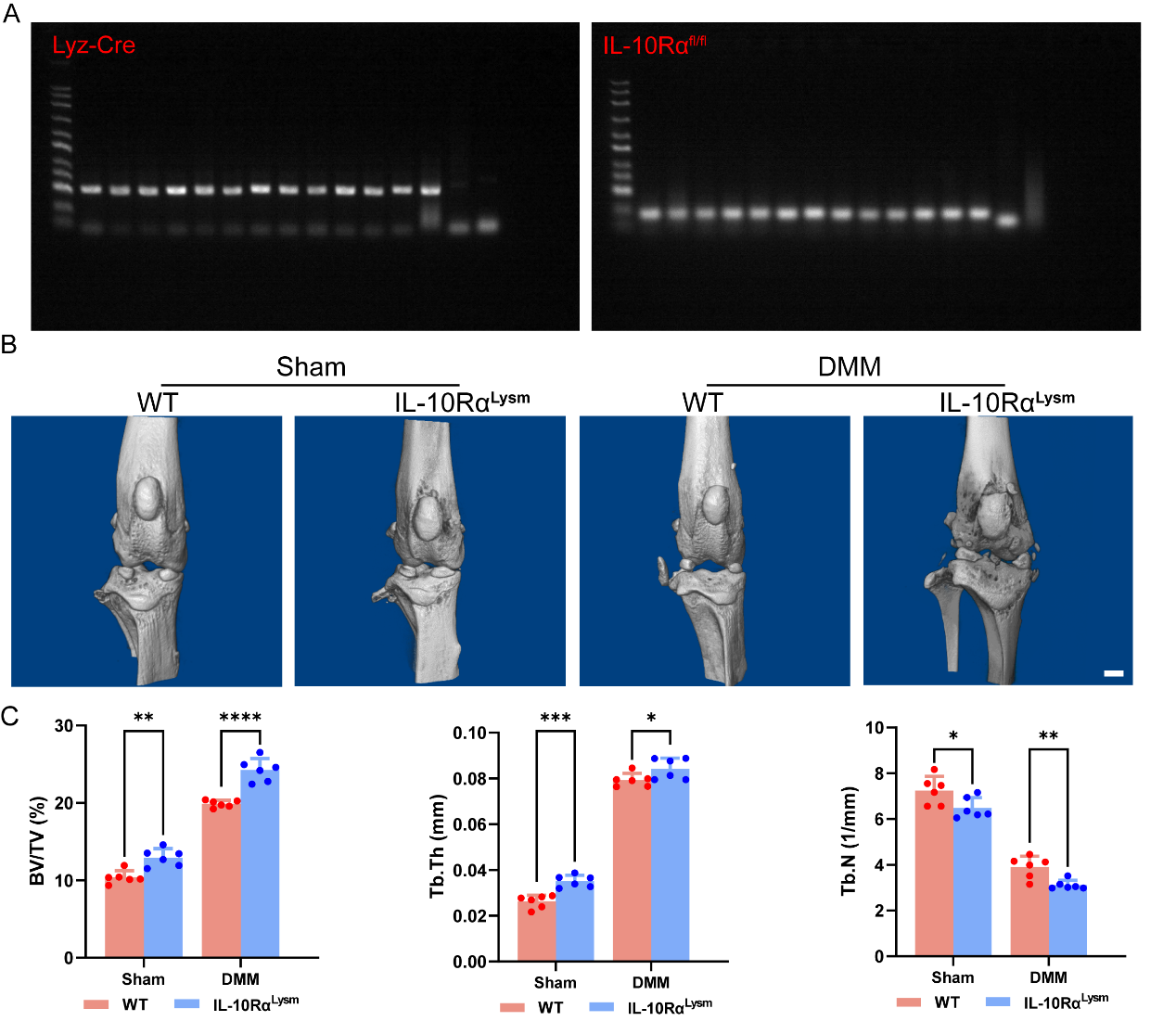


**Figure S1.** (A) Agarose gel electrophoresis results of tail DNA from IL-10Rα^Lysm^ mice. (B) Micro-CT three-dimensional imaging of the frontal view of sham and DMM mice. Scale bar: 1 mm. (C) Quantification of BV/TV, Tb.Th, and Tb.N from Micro-CT for sham and DMM mice. **p* < 0.05, ***p* < 0.01, ****p* < 0.001, *****p* < 0.001, ns indicates not significant.


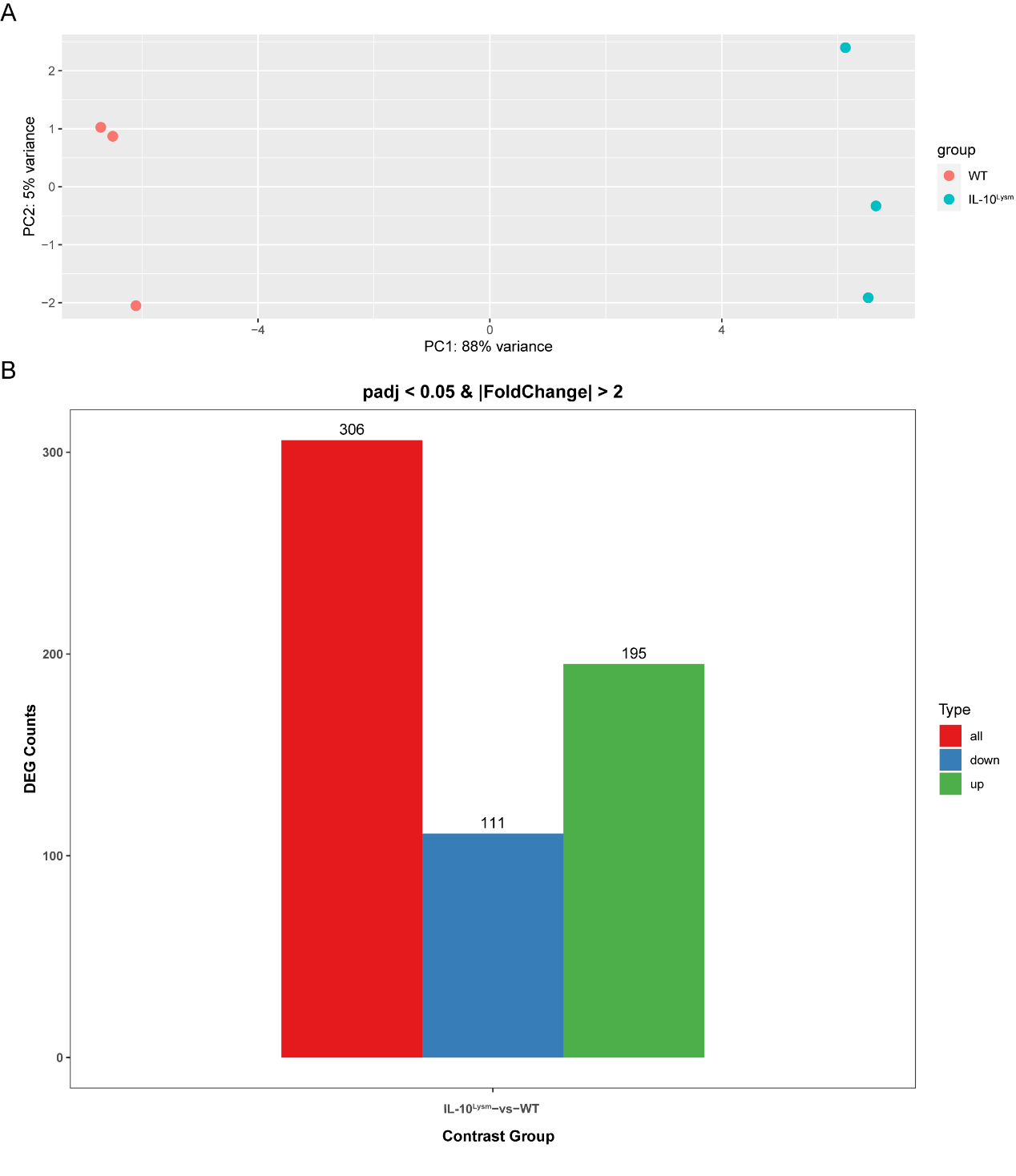


**Figure S2.** (A) Principal component analysis of samples before RNA-seq. (B) Differentially expressed genes identified by RNA-seq.


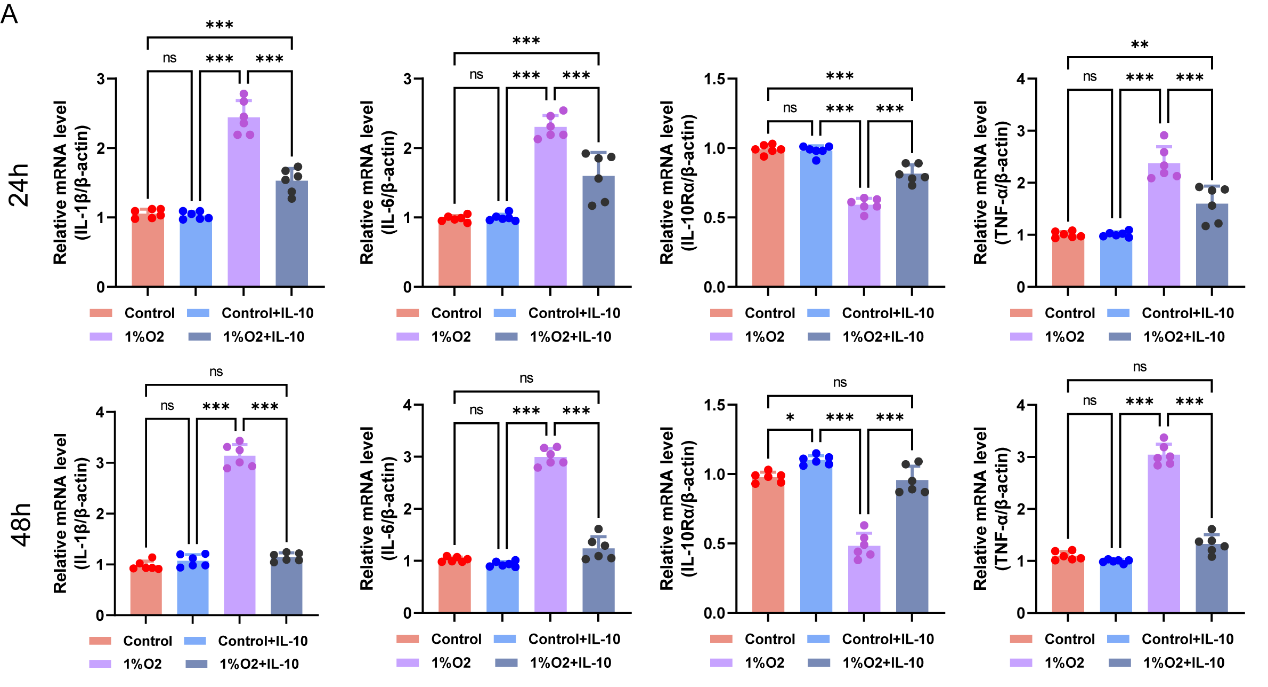


**Figure S3.** (A) qPCR results of corresponding indicators at 24 and 48 hours after the induction of hypoxia in macrophages and the addition of IL-10. **p* < 0.05, ***p* < 0.01, ****p* < 0.001, *****p* < 0.001, ns indicates not significant.


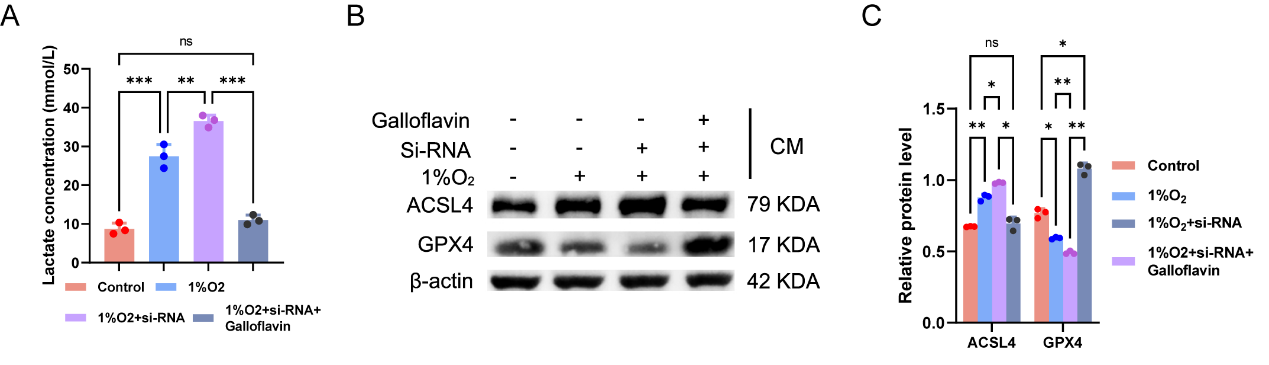


**Figure S4.** (A) Detection of lactic acid concentration in macrophage supernatant. (B, C) Chondrocytes were treated with CM, and the expression of ferroptosis-related proteins in chondrocytes was determined, along with quantification graph.

**
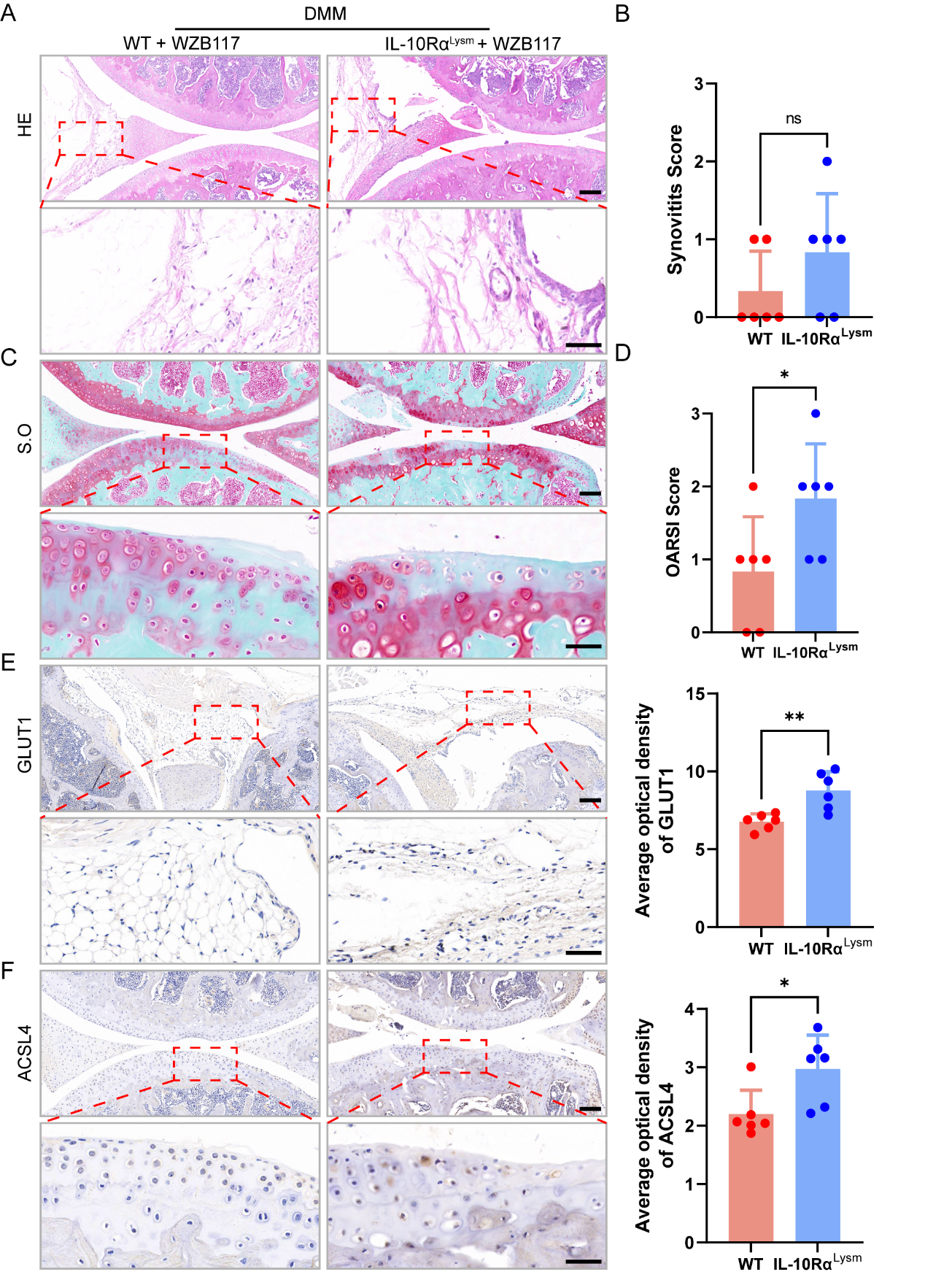
**

**Figure S5.** (A) HE staining of synovial tissue from the groups of mice. Scale bar: 100 μm. (B) Quantification of the synovitis score of the synovial tissue. (C) S.O. staining of cartilage tissue. Scale bar: 100 μm. (D) Quantification of the OARSI score. (E) IHC staining of GLUT1 in synovial tissue and quantification of positive areas. Scale bar: 100 μm. (F) IHC staining of ACSL4 in cartilage tissue and quantification of positive areas. Scale bar: 100 μm.

**
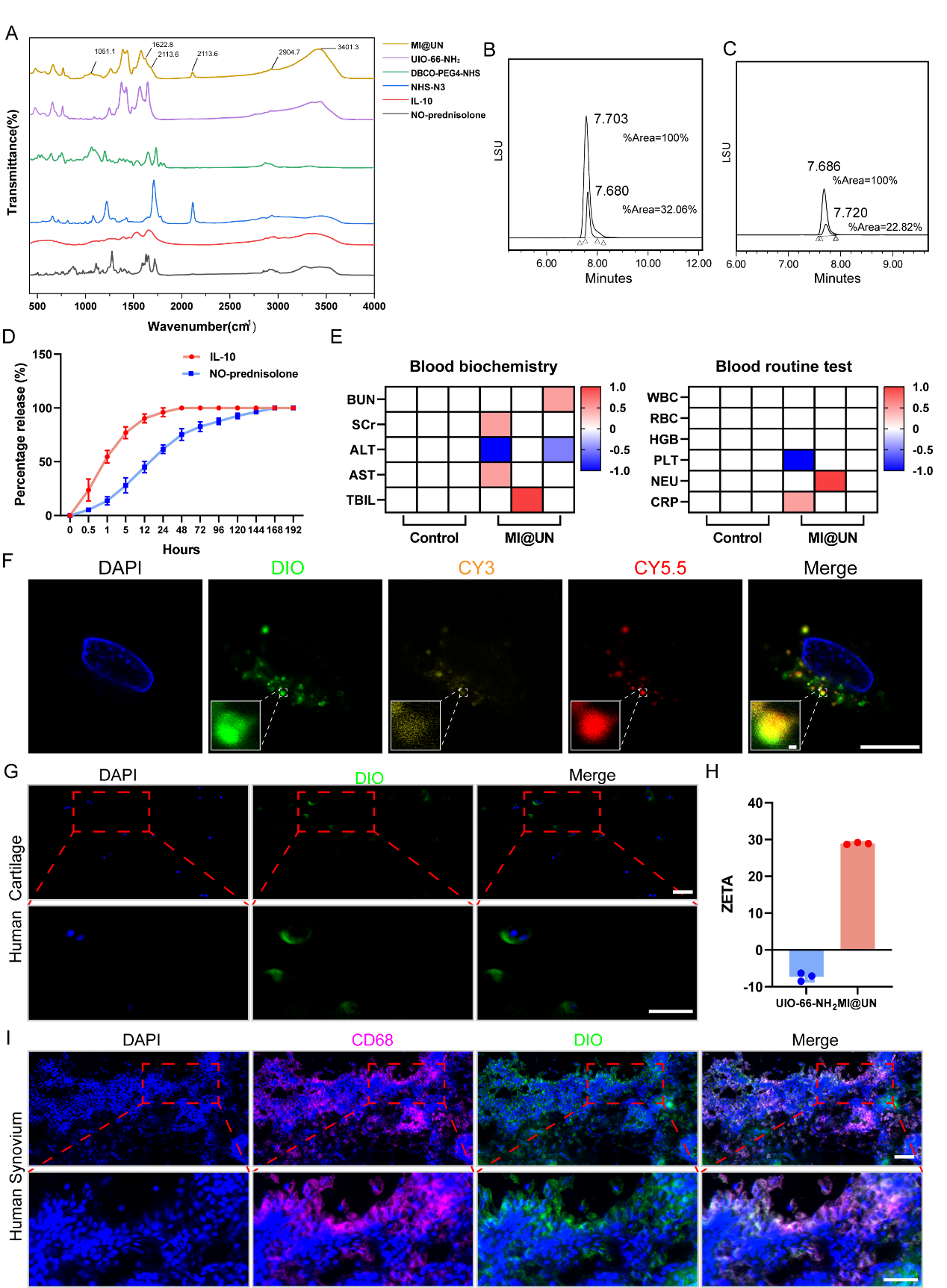
**

**Figure S6.** (A) The FITR spectra of components of MI@UN. (B) HPLC results of NO-prednisolone before and after loading. (C) HPLC results of IL-10 before and after loading. (D) In vitro release of IL-10 and NO-prednisolone from MI@UN. (E) Blood biochemical and blood routine test indicators of mice in each group. (F) Phagocytosis of MI@UN by chondrocytes, with nuclei stained with DAPI, macrophage membranes stained with DIO, IL-10 stained with CY3, and NO-prednisolone stained with CY5.5. Scale bar: 20 nm. (G) MI@UN in vitro targeting of human cartilage. Scale bar: 50 μm. (H) The ZETA potential of UIO-66-NH_2_ and MI@UN. (I) MI@UN in vitro targeting of human synovial membrane. Scale bar: 100 μm.


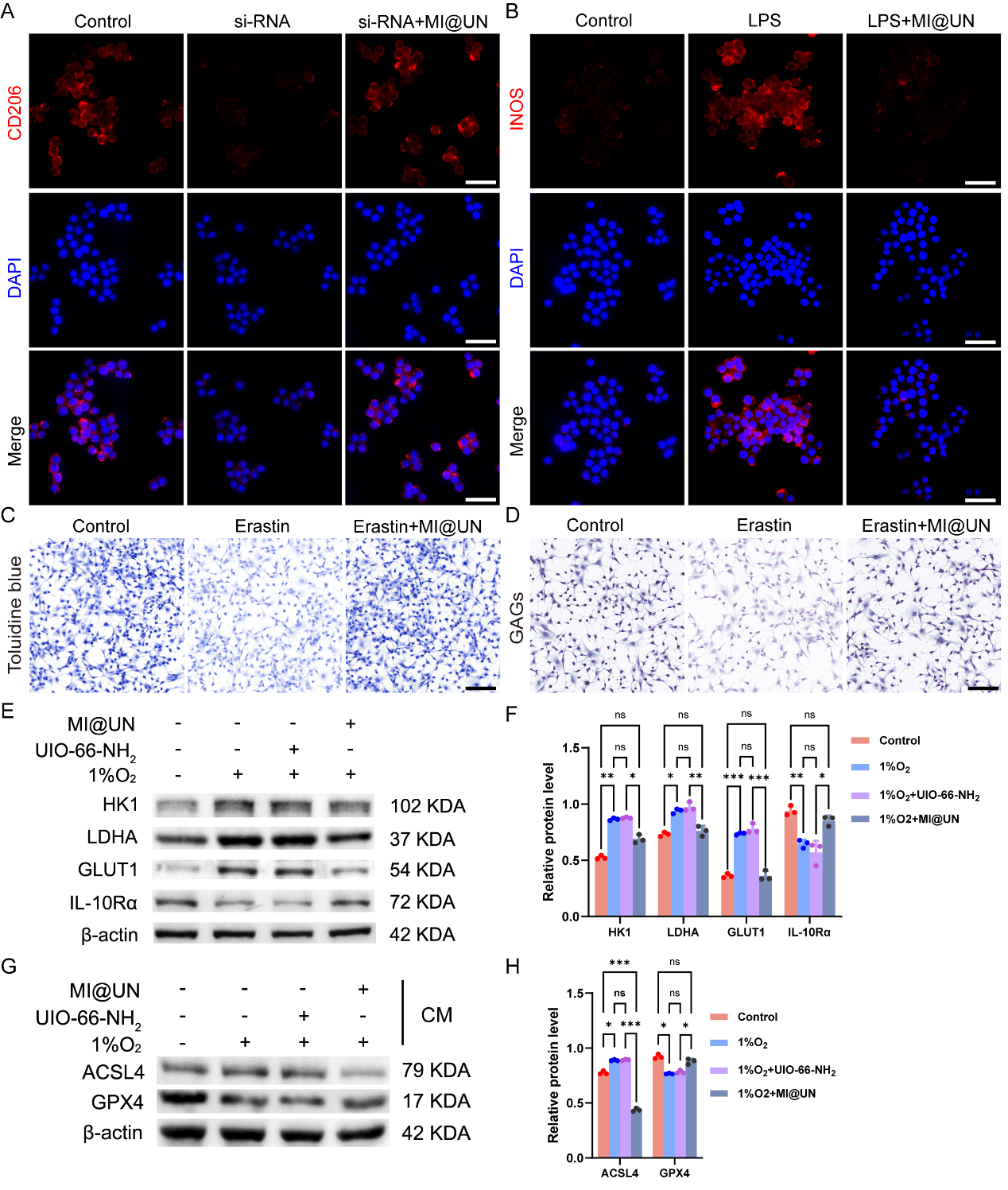


**Figure S7. (A) MI@UN has a protective effect on macrophages and promotes M2 polarization of macrophages.** Scale bar: 50 μm. **(B) The protective effect of MI@UN on macrophages, inhibiting macrophage M1 polarization.** Scale bar: 50 μm. **(C) Toluidine blue staining demonstrated the protective effect of MI@UN on chondrocytes.** Scale bar: 100 μm. **(D) Glycosaminoglycans (GAGs) were stained to demonstrate the protective effect of MI@UN on chondrocytes.** Scale bar: 100 μm. (E, F) Effect of MI@UN on the glycolysis of macrophages by WB results and quantification. (G, H) Effect of CM containing MI@UN in macrophages on ferroptosis of chondrocytes and quantification.


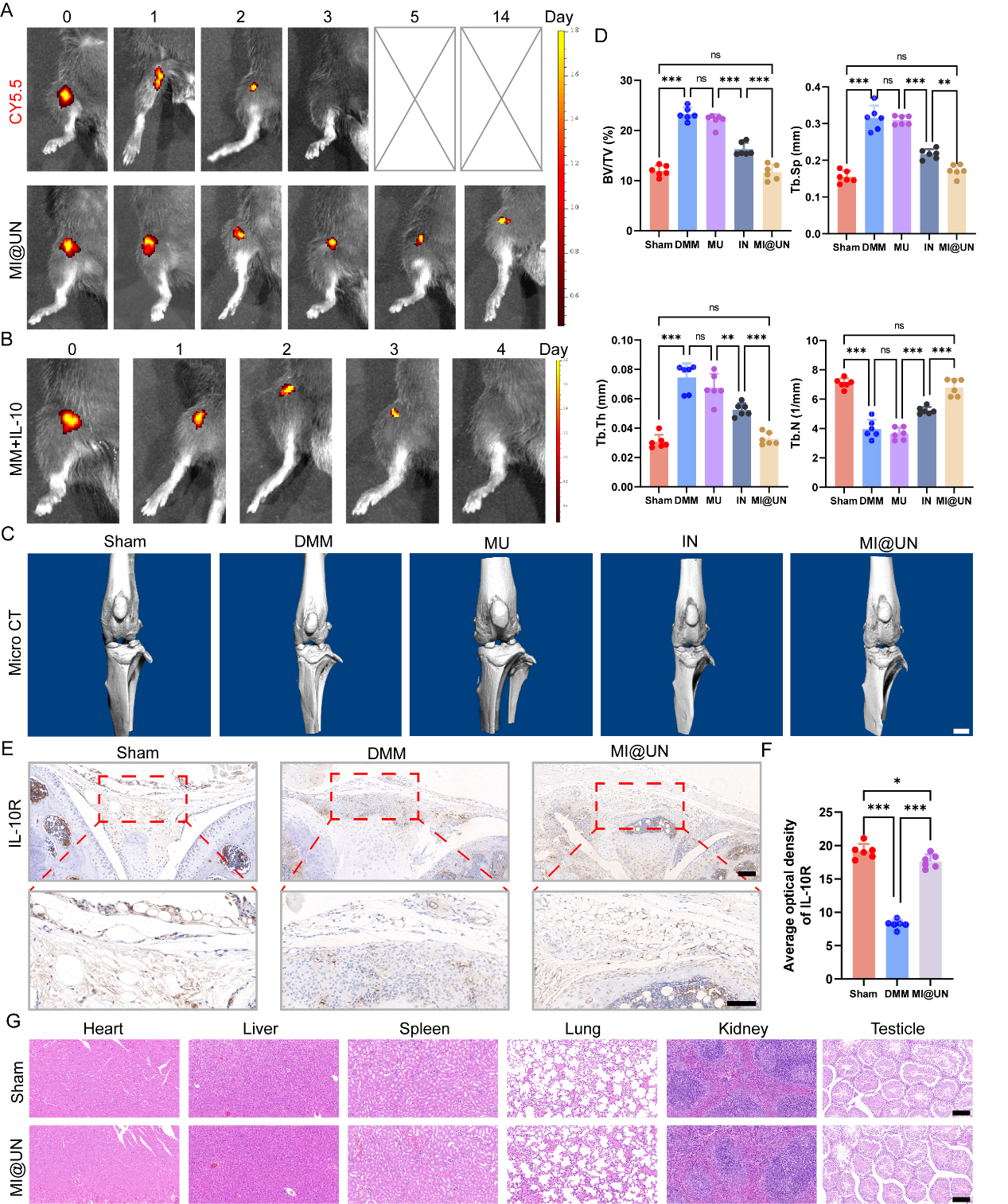


**Figure S8.** (A) In vivo imaging showing the retention time of CY5.5-labeled IL-10 and NO-prednisolone in the mouse joint cavity compared with the retention time of MI@UN in the joint cavity. (B) In vivo imaging showing the retention time of IL-10 coated with MM in the joint cavity. (C, D) Micro-CT three-dimensional imaging of the frontal view of the mouse knee joint and quantification of BV/TV, Tb.Sp, Tb.Th, and Tb.N. Scale bar: 1 mm. (E, F) Immunohistochemical staining of IL-10R. Scale bar: 100 μm. (G) Hematoxylin and eosin staining of major organs from the sham group and MI@UN group. Scale bar: 100 μm. **p* < 0.05, ***p* < 0.01, ****p* < 0.001, *****p* < 0.001, ns indicates not significant.

**
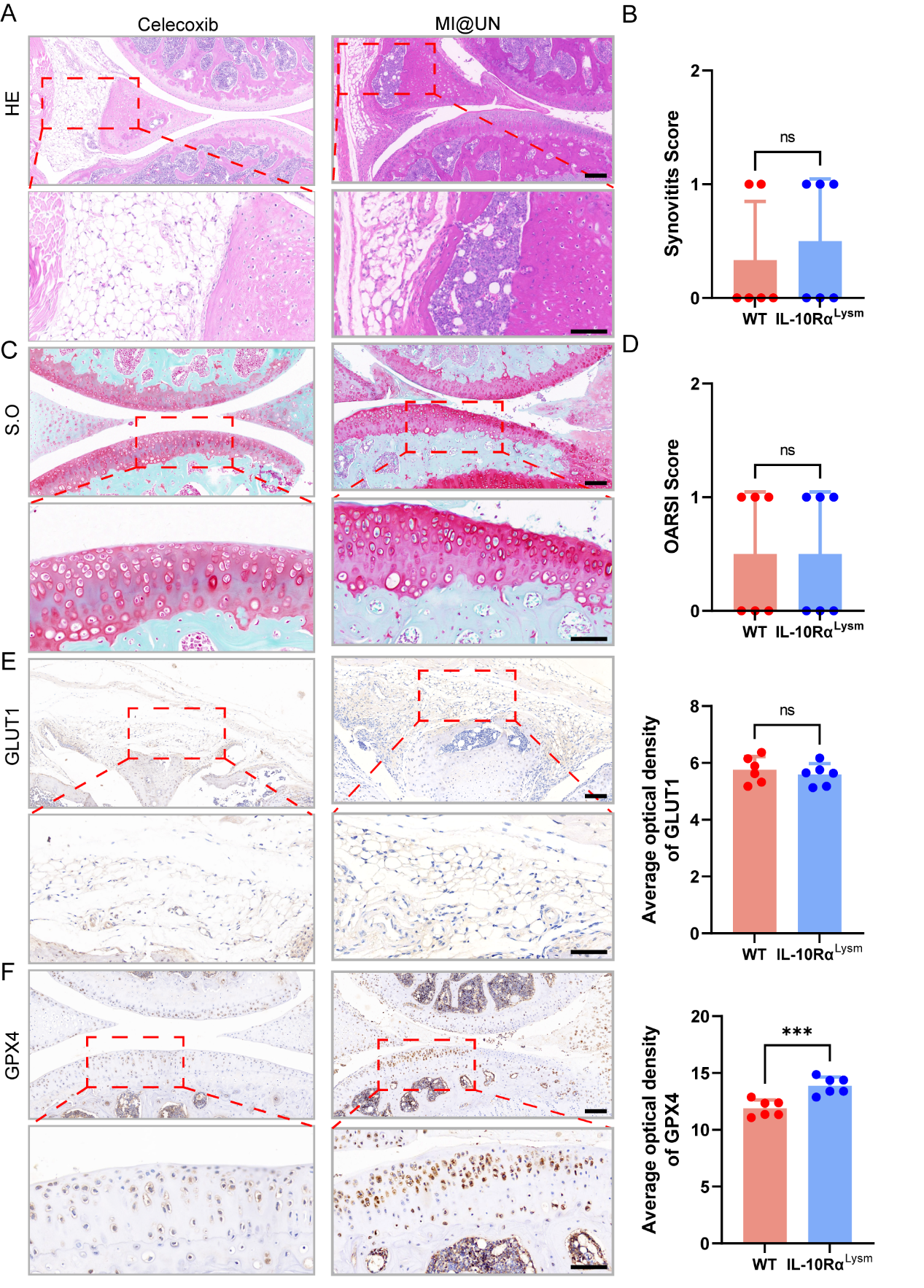
**

**Figure S9.** (A) HE staining of synovial tissue from the groups of mice. Scale bar: 100 μm. (B) Quantification of the synovitis scores of the synovial tissue. (C) S.O. staining of cartilage tissue. Scale bar: 100 μm. (D) Quantification of the OARSI score. (E) Immunohistochemical staining of GLUT1 in synovial tissue and quantification of positive areas. Scale bar: 100 μm. (F) Immunohistochemical staining of GPX4 in cartilage tissue and quantification of positive areas. Scale bar: 100 μm.


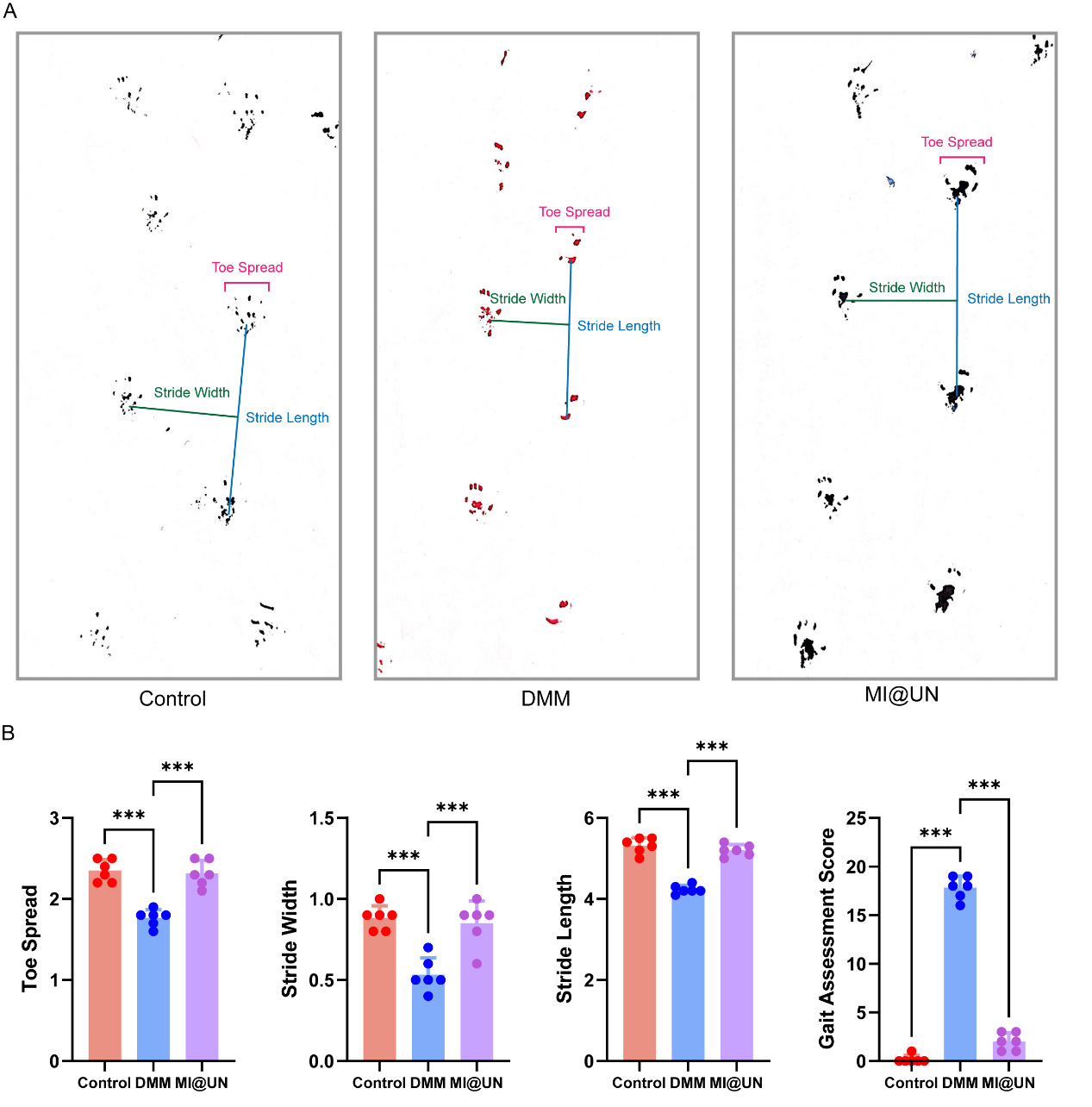


**Figure S10. (A) Gait analysis to assess the treatment of MI@UN. (B) Gait assessment scores to assess the treatment of MI@UN.**

**Table S1. Synovitis score.**

| Enlargement of the synovial lining cell layer | |
| --- | --- |
| 0 point | The lining cells form one layer |
| 1 point | The lining cells form 2–3 layers |
| 2 points | The lining cells form 4–5 layers, few multinucleated cells might occur |
| 3 points | The lining cells form more than 5 layers, the lining might be ulcerated and multinucleated cells might occur |
| Density of the resident cells | |
| 0 point | The synovial stroma shows normal cellularity |
| 1 point | The cellularity is slightly increased |
| 2 points | The cellularity is moderately increased, multinucleated cells might  occur |
| 3 points | The cellularity is greatly increased, multinucleated giant cells, pannus  formation and rheumatoid granulomas might occur |
| Inflammatory infiltrate | |
| 0 point | No inflammatory infiltrate |
| 1 point | Few mostly perivascular situated lymphocytes or plasma cells |
| 2 points | Numerous lymphocytes or plasma cells, sometimes forming follicle‐  like aggregates |
| 3 points | Dense band‐like inflammatory infiltrate or numerous large follicle‐like  aggregates |
| Sum | |
| 0-1 | No synovitis |
| 2-4 | Low‐grade synovitis |
| 5-9 | High‐grade synovitis |

**Table S2. Osteoarthritis Research Society International (OARSI) scores**

| Surface intact, cartilage intact | 0 |
| --- | --- |
| Surface intact | 1 |
| Surface discontinuity | 2 |
| Vertical fissures | 3 |
| Erosion | 4 |
| Denudation | 5 |
| Deformation | 6 |

**Table S3. Antibody serial number and dilution ratio**

| Antibody | Company (Cat. # ) | WB | IF | IHC |
| --- | --- | --- | --- | --- |
| HIF-1α | 66730-1-lg, Proteintech Group | 1:2000 | 1:100 |  |
| GLUT1 | 21829-1-AP, Proteintech Group | 1:2000 | 1:100 | 1:50 |
| LDHA | 21799-1-AP, Proteintech Group | 1:5000 |  |  |
| HK1 | 19662-1-AP, Proteintech Group | 1:2000 |  |  |
| GPX4 | AB125066, Abcam | 1:1000 | 1:100 | 1:50 |
| ACSL4 | 22401-1-AP, Proteintech Group | 1:2000 |  | 1:50 |
| β-actin | 81115-1-RR, Proteintech Group | 1:2000 |  |  |

**Table S4. Primers**

| Primer | Forward | Backward |
| --- | --- | --- |
| IL-1β | 5'-GCAACTGTTCCTGAACTCAACT-3' | 5'-ATCTTTTGGGGTCCGTCAACT-3' |
| TNF-α | 5'-CCTCTCTCTAATCAGCCCTCTG-3' | 5'-GAGGACCTGGGAGTAGATGAG-3' |
| IL-6 | 5'-ACTCACCTCTTCAGAACGAATTG-3' | 5'-CCATCTTTGGAAGGTTCAGGTTG-3' |
| IL-10Rα | 5'-CAGTGTTGGGGTGTCAGTG-3' | 5'-GTAGGTCTGGGGTCTTGAGC-3' |

**Table S5. Osteoarthritis Mouse Gait Assessment Scoring Table**

| **Assessment Indicator** | **Normal (0 points)** | **Mild Abnormality (1 point)** | **Moderate Abnormality (2 points)** | **Severe Abnormality (3 points)** |
| --- | --- | --- | --- | --- |
| **Gait Frequency** | Normal gait frequency, no obvious changes | Slight decrease in gait frequency | Obvious decrease in gait frequency | Significant decrease in gait frequency, unsteady gait |
| **Stride Length** | Normal stride length, no obvious changes | Slight shortening of stride length | Obvious shortening of stride length | Significant shortening of stride length, unsteady gait |
| **Stride Width** | Stable stride width, symmetrical on both sides | Slight changes in stride width, but basically symmetrical | Obvious changes in stride width, asymmetrical | Significant changes in stride width, asymmetrical, unsteady gait |
| **Support Duration** | Normal support duration, coordinated with swing time | Slight increase or decrease in support duration | Obvious increase or decrease in support duration | Significant increase or decrease in support duration, unsteady gait |
| **Swing Duration** | Normal swing duration, coordinated with support time | Slight increase or decrease in swing duration | Obvious increase or decrease in swing duration | Significant increase or decrease in swing duration, unsteady gait |
| **Coordination** | Good coordination among homologous, ipsilateral, and contralateral limbs | Slight abnormality in coordination, but does not affect overall gait | Obvious abnormality in coordination, affecting gait stability | Significant abnormality in coordination, severely unsteady gait |
| **Limping Degree** | No limping | Mild limping, occasionally visible | Obvious limping, easily observable | Severe limping, continuously visible |
| **Joint Swelling** | No swelling | Slight swelling, not obvious | Obvious swelling, joint contour discernible | Severe swelling, joint deformity |
| **Range of Motion** | Normal range of motion, no obvious limitations | Slight limitations in range of motion | Obvious limitations in range of motion | Severe limitations in range of motion, difficulty in movement |
